# Multiplexed Biosensing of Proteins and Virions with Disposable Plasmonic Assays

**DOI:** 10.1021/acssensors.2c02238

**Published:** 2023-08-23

**Authors:** Stephanie Wallace, Martin Kartau, Tarun Kakkar, Chris Davis, Agnieszka Szemiel, Iliyana Samardzhieva, Swetha Vijayakrishnan, Sarah Cole, Giuditta De Lorenzo, Emmanuel Maillart, Kevin Gautier, Adrian J. Lapthorn, Arvind H. Patel, Nikolaj Gadegaard, Malcolm Kadodwala, Edward Hutchinson, Affar S. Karimullah

**Affiliations:** † School of Chemistry, University of Glasgow, Joseph Black Building, University Avenue, G12 8QQ Glasgow, U.K.; ‡ James Watt School of Engineering, University of Glasgow, Rankine Building, Oakfield Avenue, G12 8LT Glasgow, U.K.; § MRC-University of Glasgow Centre for Virus Research, 464 Bearsden Road, G61 1QH Glasgow, U.K.; ∥ HORIBA France SAS, 14, Boulevard Thomas Gobert-Passage Jobin Yvon, CS 45002, 91120 Palaiseau, France

**Keywords:** biosensing, disposable plasmonics, virus diagnostics, multiplexing, chiroptical

## Abstract

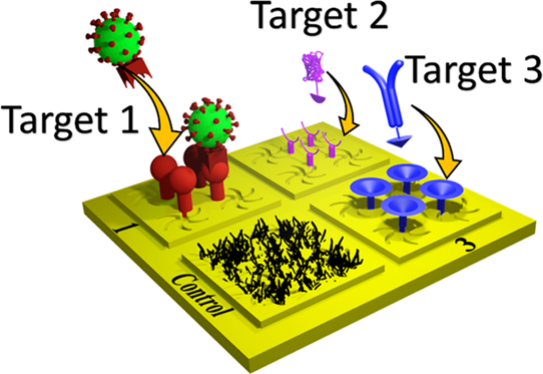

Our growing ability to tailor healthcare to the needs of individuals has the potential to transform clinical treatment. However, the measurement of multiple biomarkers to inform clinical decisions requires rapid, effective, and affordable diagnostics. Chronic diseases and rapidly evolving pathogens in a larger population have also escalated the need for improved diagnostic capabilities. Current chemical diagnostics are often performed in centralized facilities and are still dependent on multiple steps, molecular labeling, and detailed analysis, causing the result turnaround time to be over hours and days. Rapid diagnostic kits based on lateral flow devices can return results quickly but are only capable of detecting a handful of pathogens or markers. Herein, we present the use of disposable plasmonics with chiroptical nanostructures as a platform for low-cost, label-free optical biosensing with multiplexing and without the need for flow systems often required in current optical biosensors. We showcase the detection of SARS-CoV-2 in complex media as well as an assay for the Norovirus and Zika virus as an early developmental milestone toward high-throughput, single-step diagnostic kits for differential diagnosis of multiple respiratory viruses and any other emerging diagnostic needs. Diagnostics based on this platform, which we term “disposable plasmonics assays,” would be suitable for low-cost screening of multiple pathogens or biomarkers in a near-point-of-care setting.

The use of biomarkers for precision medicine allows great advancements to aid in the improvement of human health as well as the reduction in healthcare costs.^1,2^ Yet often, time, costs, and capability of current technology limit the applicability of precision medicine concepts. The COVID-19 pandemic has demonstrated a need to monitor our health more regularly.^3^ While daily testing is not an immediate necessity, a routine approach may become the norm to maintain social healthcare standards. Polymerase chain reaction (PCR) diagnostic approaches are currently the gold standard of infectious disease diagnostics, but their cost and turnaround time make them impractical for use in large-scale routine testing. PCR diagnostics are also susceptible to shortage of oligonucleotide reagents, as seen in the SAR-CoV-2 pandemic.^4^ It has also been argued that sensitivity should be secondary compared to test frequency for large-scale population testing.^5^ Modeling of the SARS-CoV-2 pandemic indicates that such a strategy is theoretically capable of reducing the reproduction “R” number of an epidemic.^5,6^

Rapid and economical detection of some pathogen components and biomarkers has been achieved using lower sensitivity tests such as lateral flow devices (LFDs) and traditional immunoassays (e.g., ELISA). LFDs, in particular, have made a significant step toward readily available mobile testing and are arguably the most cost-effective and simplest testing techniques, albeit without quantification.^3,7^ Yet, when applied to more than a single disease, these methodologies are either not high-throughput or require multiple reagents and lack ease of use. These technological limitations are a bottleneck in our progress toward being able to test rapidly for multiple pathogens with high-throughput and low costs.^3,7,8^ LFDs require multiple antibodies plus label/color-producing reagents, which often suffer from reduced sensitivity and reliability.^3,7,9^ Furthermore, the diffusion-based paper flow methodology limits the ability to add additional tests in a small area due to interference of flow paths and/or interference between sequential detection sites if positioned within a common flow path.^10^ Hence, multiple testing with LFDs either involves complicated manufacturing methods or leads to large dimensions increasing cost and reducing mobility. Consequently, LFDs are often limited to detecting a few biomarkers.^10^

Optical-based biosensing was long heralded as the best route to label-free point-of-care (PoC) multiplexed diagnostics.^11^ Using the overlap between analytical chemistry and optical sensing, plasmonic sensors can detect interactions between a monolayer of surface-immobilized binders and their target biomarkers. Due to their label-free sensing capability, the only reagents required are a buffer and the antibody/binder. A variety of plasmonic-based techniques have been implemented such as surface plasmon resonance (SPR), surface-enhanced Raman spectroscopy (SERS), localized surface plasmon resonance (LSPR), and plasmonic-based colorimetric assays.^9^ SPR-based biosensors have been the most successful with surface functionalization techniques to enhance specificity between biomarkers and surface-attached ligands and achieve diagnostics of diseases such as AIDS and hepatitis.^12,13^ They only require binder immobilization and buffers as reagents and are not dependent on adding any nanoparticles or additional steps such as mixing or rinsing. Recently, commercially available SPR biosensors have achieved portability such as systems produced by Affinite and Plasmetrix, making SPR more accessible for analytical science.^14,15^ However, high reagent volume requirements and complexity still persist.^16^ LSPR devices were supposed to mitigate these issues but have often suffered difficulties such as reduced sensitivity to refractive indices. Complex nanostructure design could potentially allow for high-quality factors and improved sensing performances but is restricted due to high manufacturing costs of consumables and reproducibility problems. A large number of LSPR sensors are still based on nanoparticles in solutions or colloidal Au-based films but have started seeing success with companies such as LamdaGen and Nicoya for the biosensor market.^16−20^ In terms of PoC diagnostics commercially, to the best of our knowledge, only Genalyte has been able to successfully use a photonics sensor to provide label-free multiplexed diagnostics, albeit still using a relatively complex and expensive consumable based on split-ring resonators on Si substrates.^21^

Herein, we report on the use of injection-molded nanopatterned polycarbonate templates, with complex nanostructure geometries, for use in multiplexed biosensing of proteins and virions as a proof of concept for the development of a multiplexed low-cost diagnostics platform. These low-cost templated plasmonic substrates (TPSs) are capable of large-scale multiplexing with high surface sensitivity, allowing for the development of multipathogen diagnostic assays that we call “disposable plasmonic assays” (DPAs). We performed label-free biosensing without any flow setup or microfluidics, demonstrating the potential of DPAs to be used as a simplified platform for PoC diagnostics. The disposable plasmonics concept has previously been used for chiral plasmonic sensing, a technique that uses chiral nanostructures with biostructural sensitivity to measure protein binding interactions. In this work, we use the chiral nanostructures for their sharp optical rotation dispersion (ORD) response with their high figure of merit (FOM) that enables better-automated peak detection and performance for label-free measurement. The chiral nanostructures used have high refractive index sensitivity (∼400 nm/RIU) and surface sensitivities as their fields decay significantly by ∼25 nm above the sensor surface. Using a hyperspectral polarimetry imaging instrument, we were able to measure multiple nanostructure arrays in a single experiment and mitigated the need for microfluidics by simply pipetting materials onto the sample surface. Through immobilization of different protein binders on the arrays, this system is capable of multiplexed label-free assays that are free of any flow systems and could therefore enable single-step testing. The measurement performance of the sensor platform was evaluated first, followed by label-free detection of protein binding events. The potential multiplexing capabilities were also demonstrated by the specific detection of antibodies for the SARS-CoV-2 spike glycoprotein S1 (anti-S1) and streptavidin (anti-streptavidin) in a single experiment with sequential addition of the targets. Lastly, the detection of SARS-CoV-2, Noro, and Zika virus (ZIKV) was demonstrated using functionalized antibodies.

## Results and Discussion

### Optical Characterization

**Figure 1:**
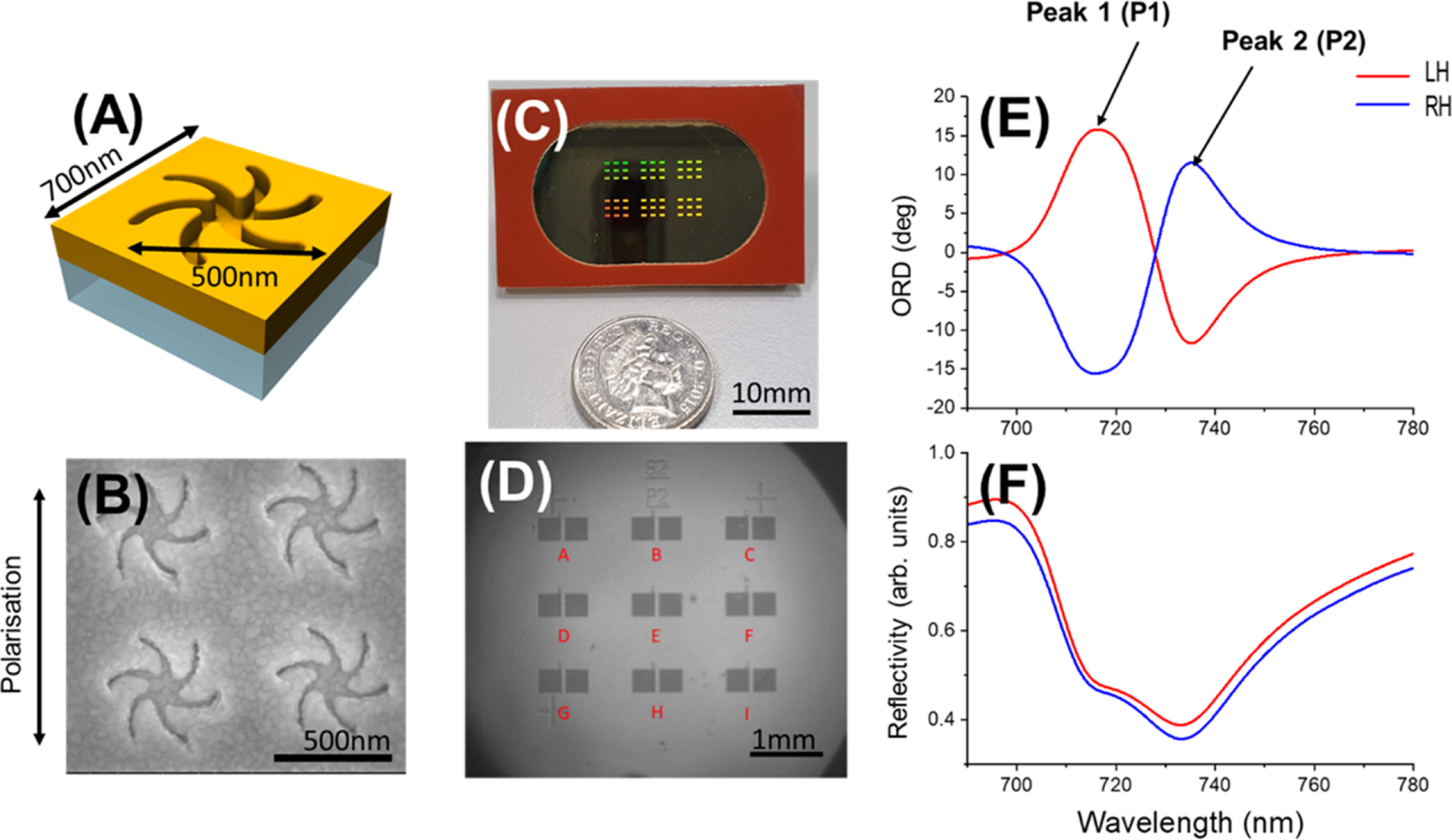
(A) Top of the TPS film with shuriken indentations, as shown in (B) scanning electron microscopy (SEM) images. (C) Sample with multiple experimentation locations compared to a 5p coin. (D) Arrays in a single experiment as viewed by the imaging instrument for multiplexing. (E) ORD (peak 1 and peak 2) and (F) reflectivity spectra produced by the biosensor.

The TPSs were generated by Au coating of an injection-molded plastic template; Figure 1C. The Au film takes on the shape of the nanostructured indentations on the plastic surface, producing a metafilm. The process has been used previously and provides a high-throughput (12 samples every 6s) manufacturing process with remarkable resolution (∼20 nm linewidths) that is similar to the manufacturing of Blu-ray disks.^22^ Specifically, we use chiral shuriken-shaped indentations as the plasmonic resonator units (Figure 1A,B) used in previous studies.^23−26^ The TPSs used here are specifically designed to work with our imaging polarimetry system that recognizes 9 locations labeled A to I (Figure 1D) for multiplexing purposes. Each location has 2 nanostructured arrays, one with left-handed (LH) nanostructures and one with right-handed (RH) nanostructures. We can use LH and RH resonance shifts either to evaluate differences for chiral plasmonic sensing or use individual resonance shifts of all 18 nanostructured arrays to gather values for our biosensing measurements. The entire measurement region is approximately a 3 × 3 mm square, and each nanostructured array has an area of 0.09 mm^2^. Solutions are added using a pipette through ports in a custom-designed fluidic chamber well, and no flow systems are incorporated into the setup (Supporting Information, Figure S1). The imaging instrument is capable of measuring ORD and reflectivity over the visible spectrum using hyperspectral imaging and polarization-dependent filters in ∼5 min. A MATLAB script automatically evaluates the ORD peaks from the measured spectra and provides peak positions and resonance shift values (Supporting Information, Figure S1).

The TPSs display bisignate ORD with 2 inflection points that we label peak 1 and peak 2; Figure 1E. They also display a “W” shaped reflectivity arising from plasmonic-induced reflectance.^26^ The ORD can be used as both a means of looking at resonance shifts as well as providing biostructural sensitivity as achieved in previous studies.^22,23^ However, biostructural sensitivity requires the surface-immobilized biomolecules to be aligned and achieve a near-homogeneous orientation over the surface. This leads to an anisotropic dielectric layer surrounding the chiral nanostructures instead of one that is isotropic and leads to a measurable asymmetry.^24,25,27^ Such constraints on the immobilization of the biomolecules can be difficult in most functionalization strategies, and this restricts the practicality of generating assays with multiple binders for multiple targets. It is also important to note that different pathogens have different physical properties. While some viruses (for example, adenoviruses or picornaviruses) are transmitted within a rigid icosahedral “capsid” assembly of proteins, others (for example, influenza viruses and SARS-CoV-2) are enclosed in an envelope of the lipid membrane with viral proteins on its surface. These “enveloped virions” are typically variable in size and shape and are flexible enough to be physically deformed, leading to a lack of consistent anisotropy in the overall structure at the metal-dielectric boundary.^28^ Achieving an immobilized layer of virions that are all well aligned to provide an anisotropic dielectric layer is not universally applicable to all virions and proteins.

However, chiral nanostructures, beyond their biostructure sensing capabilities, show improved (FOM) owing to the increased complexity in the resonance mechanism and show improved refractive index sensitivities. Such properties improve the sensing of traditional refractive index changes.^29^ Measurement of the chiral ORD response is also less susceptible to signal losses and variations generated by absorptive molecules when measuring through the sample. This can be useful given the birefringent polycarbonate substrates coated with >100 nm thick Au restrict transmission measurements. The sharper ORD peaks, such as those shown by the shurikens, improve the automation and collection of data from the experiment. Hence, our disposable plasmonic assays continue to use chiral optical properties for sensing refractive index variations to perform biosensing. Therefore, to detect binding events, we measure ORD from the shurikens by measuring reflectivity for four Stokes parameters (details in the Supporting Information). Changes in the two ORD peaks are measured as resonance shifts termed Δλ, and the value *S* that represents spacing in wavelength values between the two peaks. Δ*S* is the change in the spacing in comparison to the initial measurement and has previously been used as an additional parameter to measure protein interactions at the surface.^24,27^

We characterized the sensing performance of the TPSs with sucrose and salt solutions. The results (Supporting Information, Figures S1 and S2) showed a sensitivity value of ∼430 nm/RIU, which is between the general sensitivities of SPR (>1000 nm/RIU) and LSPR sensors (∼100 nm/RIU) and similar to those shown by nanohole films.^30−32^ Simulations of the nanostructures (Supporting Information, Figure S3) also show that the electric field intensities are reduced to <15% of the maximum at ∼25 nm from the surface, indicating that the structures have lower decay lengths than SPR (>100 nm) and similar to LSPR (∼5–10 nm) sensors, indicating high surface sensitivities similar to LSPR sensors.^17^ The electromagnetic confinement shown by LSPR and our metafilm makes them less susceptible to bulk changes due to effects like temperature changes or additional proteins expected in serum-like samples. It also potentially provides increased sensitivity to small molecules at low concentrations.^16,33^ Hence, the shuriken metafilms combine the sensing benefits of traditional LSPR and SPR biosensors.

### Biosensing

Protein-ligand interactions of streptavidin–biotin binding were measured to test the sensor. Streptavidin binds to biotin to form one of the strongest noncovalent interactions in nature.^34^ As a tetramer, streptavidin has 4 binding sites for biotin. When binding to a biotinylated self-assembled monolayer (SAM), it is likely to bind to only 1 or 2 biotin sites on the surface at any time due to the symmetry of the streptavidin structure and due to the surface density of biotin moieties immobilized in the SAM.^35^ Hence, a minimum of 2 vacant sites would be expected for additional biotin to bind to the protein. The streptavidin–biotin interaction, therefore, becomes an appropriate model system to study the performance of our sensor platform.

**Figure 2:**
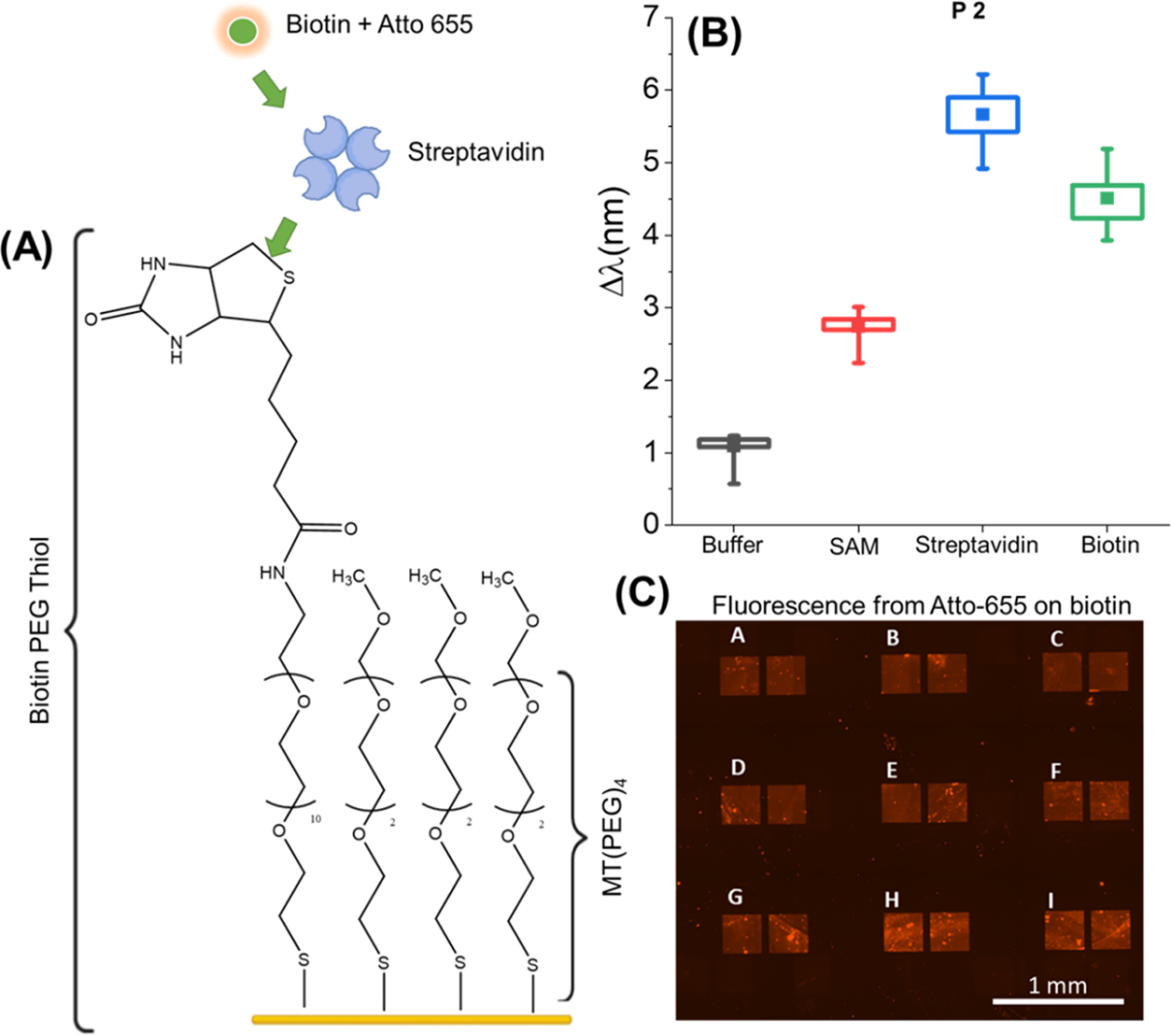
(A) Functionalization and experimental scheme: biotin PEG Thiol/MT(PEG)_4_ SAM functionalized to the Au surface; binding of streptavidin to functionalized biotin; binding of Atto-655-labeled biotin to streptavidin. (B) Results for peak 2 resonance shifts for streptavidin binding to biotin PEG thiol SAM, followed by the addition of biotin (Atto-655 conjugated). Initial measurements were taken in water and then buffer (phosphate buffer saline, PBS). Hence, biosensing measurements are taken relative to water and only Peak 2 rinsed data is shown. (C) Fluorescence from the final Atto-655 conjugated biotin observed on the nanostructures. Fluorescence over the nanostructures is more prominent due to the plasmonic enhancement.

Biotinylated poly(ethylene glycol) (PEG) thiols were immobilized with spacer molecules (methyl PEG thiol or MT-PEG) to create a SAM to functionalize the streptavidin onto the surface; Figure 2A. The spacer concentrations were optimized to completely inhibit nonspecific interactions (Supporting Information, Figure S4). Streptavidin was added for 30 min, followed by a rinse with buffer and a single measurement. The peak values were evaluated by the software from the ORD spectrum during measurement and the Δλ change from the first reference water measurements were used to plot the mean Δλ values. Figure 2B shows the resonance shifts for each peak at each step of the experiment. The box plots (25–75% quartile shown by the box and max–min range by the whiskers for all 18 nanostructured arrays) show the SAM layer shows good adhesion to the surface with a mean Δλ value of 1.6 nm for peak 2 compared to buffer. The streptavidin (∼55 kDa) is a medium-sized protein and hence generates a mean 3 nm Δλ shift (Peak 2) compared to the SAM. An additional experiment performed for the same interaction using a new sample showed good repeatability (Supporting Information, Figure S5). The streptavidin immobilization step was followed by the addition of biotin with an Atto-655 conjugate, as outlined in Figure 2A and Supporting Information Figure S6.

Upon addition of biotin, the mean resonance shifts negatively by 1.2 nm (peak 2, 40% change in value) even though biotin is bound to surface-immobilized tetrameric streptavidin as confirmed by the plasmonic enhanced fluorescence from Atto-655 conjugated to the biotin; Figure 2C.^36^ Given the extremely low dissociation constant of the streptavidin–biotin interaction, 10^–15^ M, it is highly unlikely for the biomolecules to be removed from the surface.^37^ Focusing on the mean Δ*S* values, the relatively large streptavidin causes an increase of 0.4 nm. Yet the mean Δ*S* only reduced by 0.1 nm (25% change in value) for biotin binding to streptavidin. This change in Δ*S* is far less than the change shown by the mean Δλ, contradicting streptavidin dissociation from the surface. The repeat experiment with streptavidin conjugated with Alexa Fluor 647 (results in the Supporting Information, Figure S5) showed comparable Δλ values. Hence, it can be assumed that the samples have similar amounts of streptavidin on the surface, given the same protocols for the SAM were used. While the dyes are different, fluorescence images show similar surface coverage. The results indicate that streptavidin is still bound to the surface. We hence hypothesize that the negative Δλ values would potentially be the result of structural changes (compacting) in streptavidin upon binding to additional biotin.^38−40^

### Multiplexing for Multitarget Diagnostics

**Figure 3:**
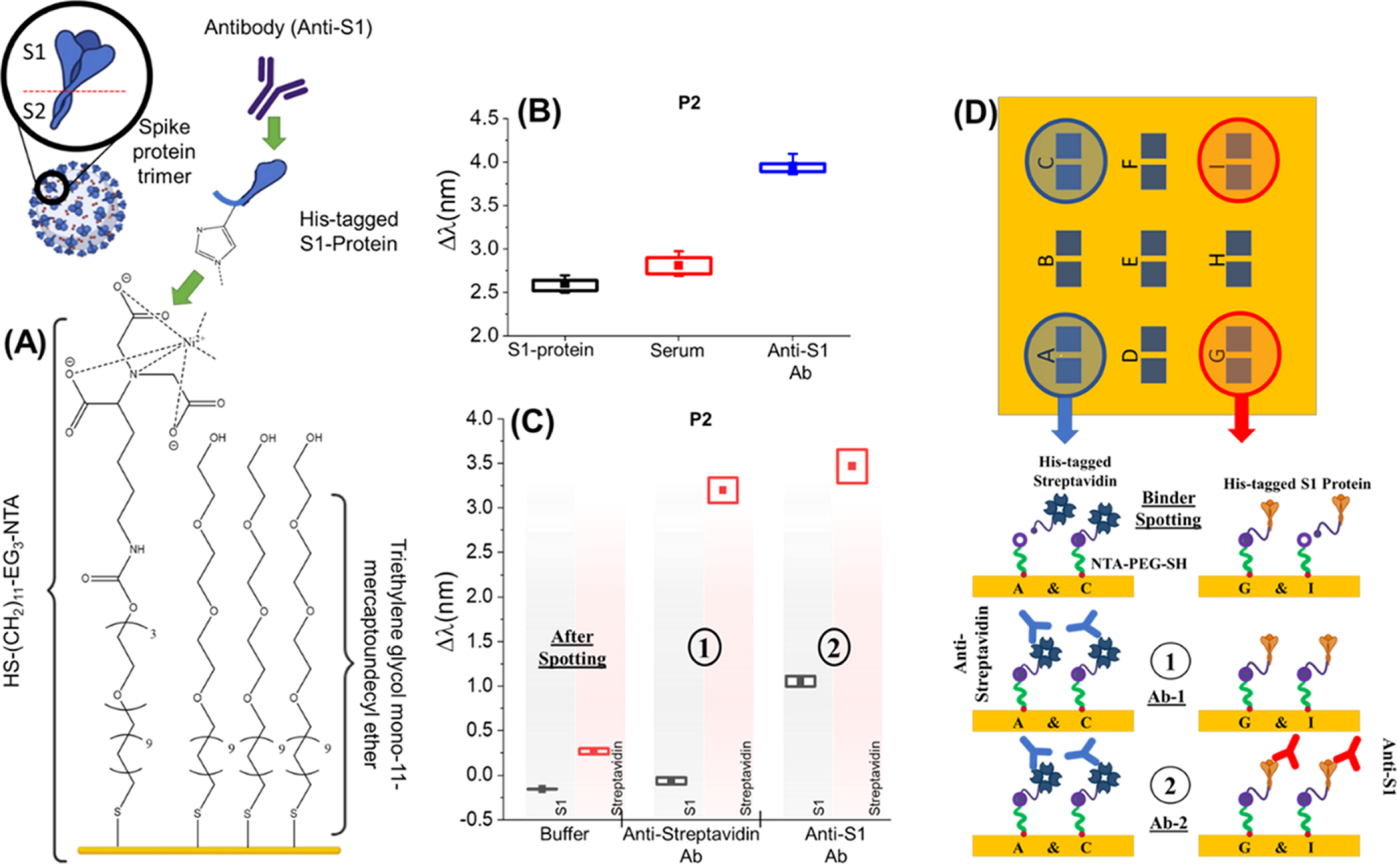
(A) Functionalization and experimental scheme of functionalized NTA/EG-thiol SAM being used to bind the His-tagged S1-protein that is then used as an antigen test to detect anti-S1 IgG antibodies. (B) Results for the 1 μM S1-protein binding to prefunctionalized NTA/EG-thiol SAM, followed by the addition of artificial mucus, which was then spiked with 1 μM anti-S1 Ab. Mean Δλ values were taken relative to water from all 18 nanostructure arrays. (C) Multiplexed DPA biosensing results showing mean Δλ values for Peak 2, with the sequential addition of target antibodies for the His-tagged S1-protein (red regions) and His-tagged streptavidin (blue regions) spotted onto prefunctionalized NTA/EG-thiol SAM. This is followed by the addition of spiked artificial mucus with (step 1) 1 μM anti-streptavidin Ab and then (step 2) 1 μM anti-S1 Ab. Results show values after rinsing steps. Each box shows a data set of all 4 nanoarrays functionalized by a single binder. (D) Graphical description of the experiment with sequential addition of the two targets.

Multiplexing in relation to biomedical diagnostics can be defined as the simultaneous measurement of multiple analytes under the same set of conditions in a single experiment and sample.^16^ A DPA antibody diagnostics proof of concept is generated by functionalizing a single TPS in four separate regions (two each) by dropping 500 nL volumes of the specific histidine (His)-tagged proteins onto TPSs coated with a SAM made using thiolated PEG with a nitrilotriacetic acid end group (NTA-PEG-thiol) and an ethylene glycol thiol (EG-thiol) spacer with a 1:4 ratio, Figure 3A. The NTA chelating agent can bind Ni^2+^, which selectively binds the His-tagged proteins. As there are no separate compartments or fluidic systems for the binders in this DPA, the target antibodies were added to the chamber sequentially to evaluate specific target recognition by monitoring the locations of the individual binders, as shown in Figure 3D.

The first of the two protein binders used was streptavidin. The second protein binder was selected to show relevance to diagnostics related to SARS-CoV-2, the virus which causes COVID-19. The virus particle is covered with a large number of glycosylated spike (S) proteins that form trimeric spikes. These are promising targets for COVID-19 antigen testing in the nasal mucus of infected individuals, and its antibody tests are useful to evaluate post-infection as well.^41,42^ We used the spike 1 (S1) protein, a subunit of the overall spike protein (details in the Supporting Information), as the binder for this purpose. The initial test of anti-S1 IgG antibody (Ab) targets binding to S1 was performed with an artificially reconstituted mimic of human mucus. Artificial mucus, termed serum, which contains 0.2% mucin, 0.25 mg/mL haptoglobin, and 0.50 mg/mL transferrin in phosphate buffer saline (PBS), showed nonspecific binding with mean Δλ increasing by 0.2 nm (peak 2, from 2.6 to 2.8 nm). This was much smaller than the specific interaction with the target anti-S1 IgG (in serum), with mean Δλ increasing by an additional 1.2 nm (to 4 nm); Figure 3B. As a reference, an additional experiment without serum was performed and is shown in Supporting Information Figure S9.

After successful confirmation of the immobilization strategy and testing the S1-protein interaction with anti-S1 Ab in artificial mucus, the multiplexed DPA was completed with streptavidin immobilized on locations A and C and the S1-protein immobilized on locations G and I; Figure 3D. All other locations were ignored. Measurements after PBS rinsing are shown in Figure 3C, where all values are the average of the two locations for each target relative to the initial buffer measurements (complete data in Figures S10 and S11 and Table S5). Each target Ab (1 μM) was spiked in artificial mucus and was introduced sequentially (step 1 for anti-streptavidin and step 2 for anti-S1) into the chamber and left for 15 min and then rinsed with PBS, after which the measurements were performed. The addition of anti-streptavidin Ab, step 1, incurred minimal nonspecific binding of this Ab to the S1-protein (change in mean Δλ is 0.1 nm (peak 2)), while specific binding to streptavidin showed a mean Δλ change of 3 nm (peak 2). Immobilization of anti-S1 Ab thereafter bound only to the S1-protein, giving a change in mean Δλ of 1.2 nm, while the streptavidin regions showed a change in mean Δλ of only 0.3 nm due to potential nonspecific interactions. These results validate the specific detection capabilities of this multiplexing setup with the potential to detect various biomolecules within one experimental setup using binders coated onto specific regions without the need for kinetic measurements or flow systems required in SPR.

It should be noted from Figure 3C that the resonance shifts exhibited for anti-S1 Ab are significantly smaller in comparison to those obtained for anti-streptavidin Ab, although both have a molecular weight of ∼150 kDa.^43^ This is likely due to the variation in coverage on the surface, hence, providing fewer epitopes for the antibodies to bind to. It is likely to be further compounded by variations in the antibody affinities for their targets.

### Detecting Virions

**Figure 4:**
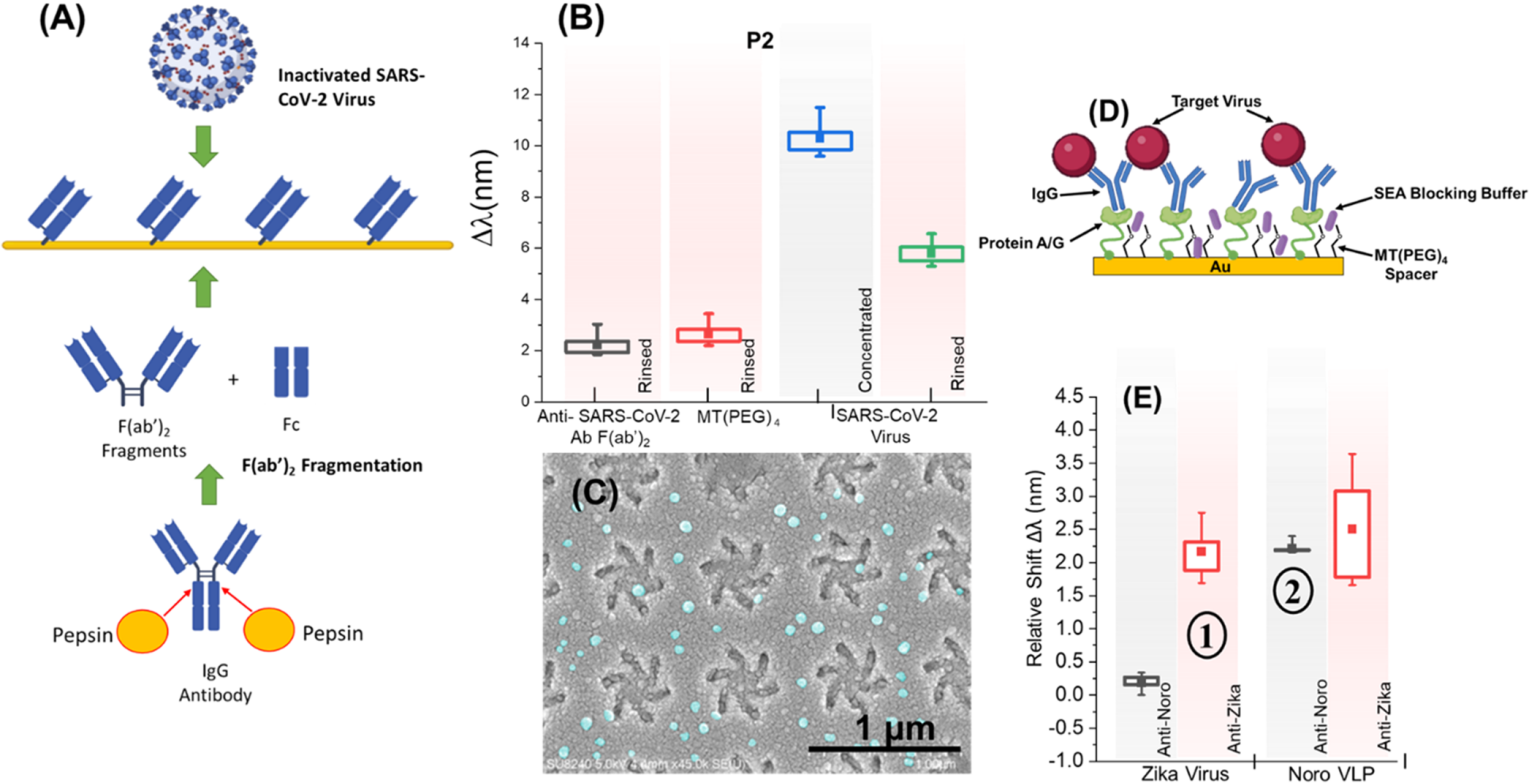
(A) Description of polyclonal F(ab′)_2_ fragmentation and its functionalization, which is then used for detecting inactivated SARS-CoV-2 Virus. The MT(PEG)_4_ spacer is not shown. (B) Results for the inactivated SARS-CoV-2 virion detection experiment. The biosensing measurements were taken relative to an initial buffer measurement. Peak 2 data are shown. (C) SEM of nanostructures with virion coverage across the surface. Prominent viruses are highlighted in a false color (cyan). (D) Scheme for the multiplexed virus DPA using cysteine terminated protein A/G, SEA blocking buffer, and specific Abs for Norovirus and ZIKV capsid/envelop proteins. (E) Relative Δλ values when Zika virions are added in step 1 and Norovirus virus-like particles (VLPs) are added in step 2 to the sample. Each step shows the specific binding of the targets to the respective regions where the gray background represents regions with anti-Norovirus and red represents regions with anti-ZIKV. Each region includes 3 sites and hence 6 nanoarrays (Supporting Information, Figure S13).

Next, we evaluated the biosensor platform for use in viral diagnostics. Additionally, we assess whether it could detect intact virions. These biological structures are substantially larger and more complex than individual proteins, and if they could be detected directly, it would remove the need for lysis of samples, reducing the processing steps and reagents required. As an example, we targeted the SARS-CoV-2 virion for our DPA. Antibodies are the classic binders used in most immunoassay diagnostics, and here, we use the anti-S1 polyclonal antibody (pAb) as the binder. However, instead of functionalizing antibodies through chemical moieties in our SAM, we use a simpler approach by immobilizing Fab′ antibody fragments directly onto the Au surface.^25^ This reduces the need for additional functionalization steps saving time and materials. SARS-CoV-2-binding antibodies were cleaved below the hinge region using immobilized pepsin. In the presence of Au, the F(ab′)_2_ fragments cleave to form F(ab′) fragments and allow direct functionalization onto the TPSs, as described in Figure 4A (further details in the Supporting Information).

The F(ab′)_2_ fragment solution (anti-SARS-CoV-2 spike glycoprotein S1 Ab) was added to the TPA sample for 2 h, followed by addition of 2 mM MT(PEG)_4_ for 2 h to completely passivate the sample, preventing any nonspecific binding of the virus, which was added thereafter for 1 h. The chamber was rinsed using buffer (PBS) and measurements were taken. Figure 4B shows the mean Δλ values for peak 2 (peak 1 in Supporting Information Figure S12). A 2.2 nm mean Δλ value was obtained for the binding of anti-SARS-CoV-2 to the DPA, with a further 0.4 nm for the MT(PEG)_4_ spacer.

The clinical isolate SARS-CoV-2-CVR-GLA-8 was amplified to a titer of 7.3 × 10^5^ plaque-forming units (PFU)/mL (details in the Methodology section). The virions were inactivated in formaldehyde for 30 min to allow safe handling, after which excess formaldehyde was quenched by diluting 1:1 with 50 mM Tris buffer resulting in the final sample with ∼3 × 10^5^ inactivated virions per mL. After the addition of the SARS-CoV-2 virus, a measurement with the viral solution was taken (concentrated) in addition to a measurement after a buffer rinse. The addition of the diluted SARS-CoV-2 solution to the DPA yielded a 7.7 nm Δλ when taking a measurement with the virus solution (concentrated) after 1 h, which was reduced by 3.8 nm upon rinsing with buffer. This reduction in the resonance shift is indicative of the removal of nonspecifically bound virions, implying that the final resonance shifts represent the specifically bound virus material on the DPA. Scanning electron microscopy (SEM) images of the sample (Figure 4C) show small spherical particles (∼50–100 nm in diameter, false-colored in cyan) on the shuriken structures, indicative of virions, further reinforcing the resonance shifts obtained post-rinsing.

Following the SARS-CoV-2 tests, multiplexed detection of virions was performed. A multiplexed virus DPA was developed for the detection of Noro and ZIKV as an exemplar. The Ab functionalization strategy implemented used a SAM incorporating protein A/G and MT(PEG)_4_ spacer molecules to bind the Fc domain of IgG Abs, as shown in Figure 4D (further details in the Supporting Information). This strategy was found to improve the mitigation of nonspecific binding between the two virus targets compared to the Ab fragmentation protocol. Different monoclonal antibodies (mAb) were introduced onto specific regions of the DPA, which were further optimized by the addition of a SEA blocking buffer to mitigate nonspecific binding. Two regions were coated specifically with mouse anti-Norovirus mAb targeting the VP1 capsid protein and mouse anti-ZIKV mAb targeting the 150-loop of the viral envelope protein (Figure S13B). Figure 4E shows the specific response from the regions where the gray column represents results from the anti-Norovirus regions, and the red columns represent results from the anti-ZIKV regions. The Δλ values are taken after a PBS rinse and are relative to when the experiment was started. In step 1, ZIKV particles (4.8 × 10^5^ PFU/mL) are introduced into the sample, and in step 2, Norovirus virus-like particles (VLPs) are introduced into the sample. Results show specific shifts for the two targets by their specific antibody-coated regions. The anti-ZIKV region shows some nonspecific interaction; however, the mean Δλ is much lower for the nonspecific behavior. The overall mean Δλ for both targets is lower in comparison to the SARS-CoV-2 experiments. Inspection of the surface coverage by the virions and VLPs showed less coverage in comparison (Supporting Information, Figure S14) and suggests the need to further optimize multiplexed virus DPAs in the future. Using Δλ data from experiments measuring dilutions of the ZIKV (Supporting Information, Figure S15), we calculated the limit of detection (LOD) to be 3.1 × 10^4^ PFU/mL for the ZIKV assay based on typical methods used for optical sensors.^44^ These values are comparable to most optical biosensors and LFDs but still lower to modern, more sensitive LFDs and electrochemical sensors based on newer methodologies.^45,46^ Comparisons are further difficult as the method of defining the LOD is also highly variable and highly dependent on the target, their antibodies, and assay methodologies. Our SARS-CoV-2 results showed much larger resonance shifts with higher virion coverage. The performance of each assay will hence depend on the Ab performance and surface coverage of the virions. Additionally, incorporating sandwich assay methodologies including the usage of nanoparticles can improve the LOD where required but will increase the number of reagents. These results conclude the capability of DPAs to be used as multivirus diagnostic tools.

## Conclusions

The concept of DPAs could provide a mass-manufacturable route to cheaper and reliable label-free biosensors for diagnostics. Our shuriken metafilms have shown attributes of LSPR and SPR sensors, and the biosensing results here prove the validity and accuracy of multiplexed plasmonic biosensing using these substrates. Our platform, based on a hyperspectral imaging instrument and the consumable TPSs, was used successfully to detect protein–protein interactions with high sensitivity to interactions at the surface. Using this platform, a simple DPA was created with multiple functionalized binders for an antibody target detection exemplar that successfully detected antibodies in complex artificial mucus-like conditions. The direct detection of virions, including multiplexed detection, without lysing or any additional labeling, was also successfully demonstrated. Through direct functionalization of the antibodies to the surface, we demonstrate further reduction of the number of reagents required to produce such assays. With increased multiplexing, DPAs can be developed for high-throughput target screening assays. Further development toward a more compact instrument will provide PoC capability with single-step drop testing DPAs for analytical work in laboratories or in-field diagnostics for multiple respiratory viruses, saving costs and improving healthcare. This work lays the foundation of this new technological platform that can provide a seamless transition from research to in-field application with the potential to alter the way modern diagnostics and precision medicine are practiced.

## Methodology

### Fabrication of Templates

The TPSs used were generated by first producing polycarbonate templates. These templates are created by first writing a pattern in PMMA using electron beam lithography (Raith) and then electroplating to generate a Ni shim. The shim is used as the master in the injection molding to produce polycarbonate microscope slides with nanoindented surfaces that are the templates for the plasmonic metafilms. These templates are coated with a thin layer of gold (120 nm) through electron beam deposition (MEB-550s Plassys) to provide the metafilm that completes the TPSs. Each TPS is then cleaned in a 25 Watt plasma oxygen asher for 30 seconds prior to any functionalization. A customized fluidic well (GraceBio) with a glass cover slip is attached to the surface of the TPSs for experimentation. Additional details of the fabrication can be found in previous work by Stormonth-Darling et al.^47,48^

### Optical Measurements

A custom-built microscope is used to image the TPS surfaces using a CMOS camera (FLIR) with a variable polariser and a monochromatic light source (Spectral Photonics) polarized using a nanoparticle polariser (Thorlabs). More details can be found in the Supporting Information. LabVIEW software is used to control the light source wavelength and capture data from 18 locations in the image, corresponding to the nanoarrays on the TPSs, and generate the dispersion spectrums. The software calculates the peak positions, and a table is generated for all 18 locations to provide resonance peak wavelength values. Samples were placed on a stage with multiaxis alignment features, and alignment was performed to achieve even illumination and ORD with equal and opposite graphs from both left-handed and right-handed nanostructure arrays. For each experimental step, 3 measurements were taken (of all nanopatterned arrays) and the average was used to measure the resonance position for each location. Graphs for each step were produced using the mean and standard deviation for all 18 locations.

### Streptavidin Experiments

Solutions for the self-assembled monolayer (SAM) functionalization were prepared using a 1:4 ratio of Biotin(PEG) Thiol:MT(PEG)_4_ Thiol (Polypure 41156-1095; ThermoFisher 26132) with the constituents having a total 100 mM concentration in phosphate buffer saline (PBS, ThermoFisher). The sample was incubated in this solution for 24 h, followed by rinsing with PBS and measurements with PBS for the starting reference values. Streptavidin (ThermoFisher 21122) at 1 μM was prepared in PBS and added to the sample for 2 h. Measurements were taken before and after PBS rinsing. 1 μM Atto-655-Biotin (Sigma-Aldrich 06966) was also prepared in PBS and added to the sample for a further 2 h period. Again, measurements were taken pre- and post-rinsing using PBS.

### S1-Protein Experiments

The SAM was prepared using a 1:4 ratio of HS-(CH_2_)_11_-EG_3_-NTA:HS(CH_2_)_11_(OCH_2_CH_2_)_3_OH (Prochimia TH007-002; Sigma-Aldrich 673110), with the constituents having a total concentration of 1 mM in 95% ethanol. Following 4–5 h incubation of the samples in this solution, they were rinsed with 95% ethanol and incubated for a further 5 min in 1 mM aqueous NaOH. The samples were then rinsed with water and incubated in 40 mM NiSO_4_ for 1 h. Finally, the samples were rinsed with HEPES buffered saline (HBS) and water and dried with nitrogen. The fluidic chamber was attached and an initial reference measurement was taken of the SAM following rinsing with PBS and Tween20 (surfactant rinsing solution). The 125 kDa recombinant human coronavirus SARS-CoV-2 spike glycoprotein S1 (Abcam ab273068) was prepared at a concentration of 0.2 μM in PBS and applied to the sample for 1 h. Measurements were taken with the anchored S1 on the surface pre- and post-rinsing. A solution of 0.2% (w/v) mucin from bovine submaxillary glands (Sigma-Aldrich M3895), 0.25 mg/mL haptoglobin (Merck Sigma-Aldrich #H3536), and 0.50 mg/mL transferrin (Merck Sigma-Aldrich T3309) artificial mucus was prepared in PBS and added to the sample for 15 min and biosensing measurements taken. 0.2 μM anti-SARS-CoV-2 spike glycoprotein S1 mAb (Abcam ab275759) in artificial mucus was applied to the sample for 1 h. The protein–protein interaction was measured pre- and post-rinsing.

For the multiplexing setup, the SAM was prepared in a 1:4 ratio as before. A 1 μM solution of the S1-protein (Abcam ab273068) in PBS was prepared, and 0.5 μL was spotted onto specific regions of the TPSs. Recombinant His-tagged streptavidin (Prospec Pro-621) was also prepared at 1 μM in PBS and spotted onto another two regions of the TPSs. These solutions were left on the sample for 1 h, followed by PBS and Tween20 rinsing. A fluidic chamber was then attached and measurements were performed. A 1 μM anti-streptavidin antibody (Sigma-Aldrich S6390) was prepared in PBS and added to the sample for a 1 h period, followed by the addition of the 1 μM anti-S1 antibody (Abcam ab275759) for a further 1 h. Measurements were taken for both antibodies pre- and post-rinsing.

### Isolation of the SARS-CoV-2 Virus from the Clinical Sample

The SARS-CoV-2-CVR-GLA-8 virus (the clinical isolate GLA-8) was isolated from nasal swabs from SARS-CoV-2-infected individuals. The sample was isolated by co-author Chris Davis from a patient with the consent given to the ISARIC4C consortium (https://isaric4c.net/). The ethical approval for sample collection and isolation was given by the Scotland A Research Ethics Committee (reference 20/SS/0028). The samples were transported in viral transport medium (VTM) mixed 1:4 in Dulbecco’s modified Eagle medium (DMEM) supplemented with 2% fetal calf serum (FCS), 1% penicillin–streptomycin and 250 ng/mL Amphotericin B (ThermoFisher Scientific, cat# 10566016, 10499044, 15140122, and 15290018, respectively). The mixture was clarified at 3000 rpm for 10 min and then used to inoculate Vero E6 cells (African Green monkey kidney cell line from Michelle Bouloy, Institute Pasteur, France) in a 6-well plate. Samples were harvested between 48 and 96 h post-infection, depending on the extent of the cytopathic effect (CPE). The viral presence was determined using an NEB Luna Universal Probe One-Step RT-qPCR Kit (New England Biolabs, E3006) and 2019-nCoV CDC N1 primers and probes (IDT, 10006713) and infectious titers by the plaque assay. The viral sequence and the purity of the primary isolate were assessed using metagenomic next-generation sequencing. Briefly, RNA was extracted from the culture supernatant using a standard hybrid Trizol-RNeasy protocol (Thermofisher Scientific cat #15596018). Library preparations were completed from cDNA using a Kapa LTP Library Preparation Kit for Illumina Platforms (Kapa Biosystems, cat #KK8232). The sequencing of the libraries was carried out on Illumina’s NextSeq 550 System (Illumina, cat# SY-415-1002). The resulting viral stock was designated CVR-GLA-8 (Genbank accession ON911332).

A virus working stock of CVR-GLA-8 was grown on A549-ACE2-TMPRSS2 and Vero E6 cell lines, as described previously.^49^ The cells were maintained in DMEM-Glutamax supplemented with 10% fetal calf serum (FCS; Gibco) and nonessential amino acids (NEAA; Gibco) at 37 °C in a 5% (v/v) CO_2_ humidified incubator. Infections were carried out with SARS-CoV-2-CVR-GLA-8 in monolayers of the Vero E6 cells in a medium supplemented with 2% FCS and incubated at 32 °C for 7 days after which medium containing the infectious virus was harvested. To assess the infectious titer, A549_ACE2_TMPRSS2 or Vero E6 cells in 12-well plates were infected with 10-fold dilutions of virus samples. After 1 h incubation at 37 °C, 1 mL of overlay comprising MEM, 2% FCS, 0.6% Avicel (Avicel microcrystalline cellulose, RC-591) was added per well and incubated at 37 °C for 3 days. Cell monolayers were fixed with 8% formaldehyde, and plaques were visualized by staining with 0.1% Coomassie brilliant blue (BioRad cat #1610406) in 45% methanol and 10% glacial acetic acid. The CVR-GLA-8 stock titer on A549_ACE2_TMPRSS2 cells was 7.3 × 10^5^ PFU/mL and 5.3 × 10^4^ PFU/mL on Vero E6 cells.

### SARS-CoV-2 Inactivated Virus Experiments

Anti-SARS-CoV-2 spike glycoprotein S1 pAb (Abcam ab275759) was cleaved using a Pierce F(ab′)_2_ Micro Preparation kit (Thermofisher 44688) following manufacturer instructions, with the estimated antibody fragmentation being between 50 and 70%. Concentration calculations assume 50% conversion. Following this preparation, the F(ab′)_2_ fragment solution was added for 1 h, and a measurement was taken (concentrated). A 2 mM MT(PEG)_4_ spacer solution was prepared in PBS and was added for 1 h. Measurements were then taken pre- and post-rinsing with PBS.

The virus was inactivated by the addition of 0.2 mL of formaldehyde (Fisher Scientific cat #F/1501/PB17) to 1 mL of virus (final formaldehyde concentration 6% (v/v)). After 30 min incubation at room temperature, the inactivated virus solution was removed from the CL3. The inactivated virus was stored at −20 °C until further use. The inactivated virus was diluted 1:1 with TRIS buffer to quench the formaldehyde prior to application. The final solution was added to the fluidic chamber for 1 h, and measurements were taken prior to and after rinsing with PBS.

All live virus procedures were performed in a Biosafety level 3 laboratory at the MRC-University of Glasgow Centre for Virus Research (SAPO/223/2017/1a).

### Isolation of the Zika Virus from the Clinical Sample

The Zika virus strain PE243, initially isolated from a clinical source, was propagated in Vero E6 cells and its infectious titer was determined by the plaque assay as described.^50^

### Isolation of a Mouse Monoclonal Antibody to the ZIKV Envelope Glycoprotein

Balb/c mice were immunized with a synthetic peptide corresponding to the 150-loop (amino acids 144 to 166) of the ZIKV envelope protein and a monoclonal antibody (mAb), named ZkE3, was isolated using standard hybridoma technology. The specificity of mAb ZkE3 to the viral envelope 150-loop was confirmed by western blot and ELISA (data not shown). A detailed characterization of this mAb will be presented elsewhere. mAb ZkE3 was purified using protein G affinity chromatography for use in the experiments described.

### Multiplexed Virus Experiments

The SAM was prepared using a 1:30 ratio of the protein A/G Cys-tagged recombinant protein (Prospec pro-1928, concentration of 6.3 μM) and MT(PEG)_4_ spacer (189 μM, Thermofisher 26132) in ultrapure water. Following an incubation time of 16 h, the sample was rinsed with PBS. The mouse anti-Norovirus GI antibody (NativeAntigen MAB12495-100) at 1 μM and the anti-ZIKV virus antibody mAb ZkE3 at 1 μM were prepared in PBS and added to separate regions of the sample for 2 h using culture well inserts (Ibidi) to isolate the regions. The sample was rinsed with PBS, and SEA blocking buffer (Thermo 37527) was added to the sample for 30 min. The sample was rinsed with PBS, a fluidic chamber was attached, and measurements were performed. Medium containing 1.6 × 10^6^ PFU/mL ZIKV was diluted 70:30 (4.8 × 10^5^ PFU/mL) and added to the sample for 1 h. The sample was rinsed with PBS and measurements were performed. A Norovirus VLP solution (NativeAntigen REC31722-100) was added to the sample for 1 h. The sample was rinsed with PBS and measurements were performed.

## Supporting Information Available

Multiplexed biosensing of proteins and virions with disposable plasmonic assays; contents including microfluidic well setup and instrumentation; simulation for maximum electric field intensities; additional biosensing data; additional data on multiplexing experiment for multitarget diagnostics; additional data on the multiplexing experiment for multitarget diagnostics; using antibodies to detect inactivated SARS-CoV-2 virus with plasmonic sensors; additional information on multiplexed DPA for virus detection; and limit of detection (LoD) (PDF)
