# Supplementary material for: Multiplexed Biosensing of Proteins and Virions with Disposable Plasmonic Assays
Source: ACS Sens. 2023 Aug 23;8(9):3338–48. doi: 10.1021/acssensors.2c02238 (PMC10521139; doi:10.1021/acssensors.2c02238)
Supplement: Supplementary file 1 — se2c02238_si_001.pdf [file se2c02238_si_001.pdf]

Multiplexed Biosensing of Proteins and Virions with Disposable Plasmonic Assays

Stephanie Wallace,[1] Martin Kartau*,[1] Tarun Kakkar,[1] Chris Davis,[3] Agnieszka Szemiel,[3] Iliyana Samardzhieva,[1] Swetha Vijayakrishnan,[3] Sarah Cole,[3] Giuditta De Lorenzo,[3] Emmanuel Maillart,[4] Kevin Gautier,[1] Adrian J. Lapthorn,[1] Arvind H. Patel,[3] Nikolaj Gadegaard,[2] Malcolm Kadodwala,[1] Edward Hutchinson,[3] Affar S. Karimullah*.[1]

1. School of Chemistry, University of Glasgow, Joseph Black Building, University Avenue, G12 8QQ, Glasgow, UK
2. James Watt School of Engineering, University of Glasgow, Rankine Building, Oakfield Avenue, G12 8LT, Glasgow, UK
3. MRC-University of Glasgow Centre for Virus Research, 464 Bearsden Road, G61 1QH, Glasgow, UK
4. HORIBA France SAS, 14, Boulevard Thomas Gobert - Passage Jobin Yvon, CS 45002 – 91120, Palaiseau, France

Microfluidic well setup and instrumentation

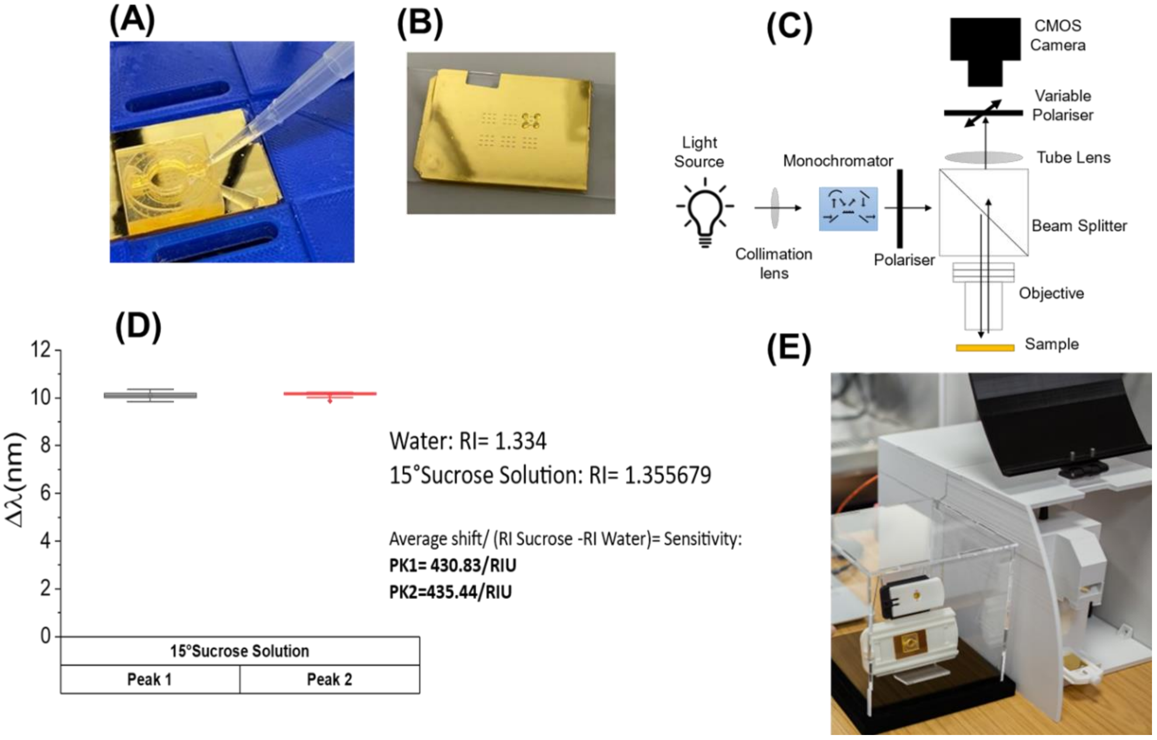

Supplementary Figure S1: (A) Solutions added to sample through a microfluidic well using a pipette. (B) Multiplexing arrangement showing spotting on the corner array sets. (C) Instrument layout for hyperspectral polarized measurements. (D) Results of sucrose solution measurements to measure the refractive index sensitivity. (E) PoC demonstrator and cartridges being exhibited.

The measurement instrument, Supplementary Figure S1 (C and E), uses a variable polariser to capture 4 images at polarisation states corresponding to 0°, 45°, 90° and 135° angles. The samples are illuminated

using a tungsten halogen light source attached to a monochromator. The monochromatic light passes through a polariser to generate a linearly polarised monochromatic light to excite the nanostructures. Images are then captured at each wavelength point. The images are then analysed in the Labview software to separate out the 18 regions and calculates the average intensity of the pixels for each array region, for each polarisation state. Using Stokes equation (below), the optical rotation can then be calculated and plotted for each wavelength producing the ORD plot.

$$ORD = \frac{1}{2} tan^{-1} \frac{(I_{45} - I_{135})}{(I_0 - I_{90})}$$

Where I represents the intensity and the subscript represents the polarisation angle. The monochromator steps by 1nm for each wavelength point and the entire spectrum is measured in ~5mins. The software performs peak detection using the ORD spectrum and stores the resonance wavelength position for each nanoarray.

The microfluidic chamber (Supplementary Figure S1 (A)) is customised to adhere directly to the gold surface of the sample. A handheld pipette is used to inject 100 uL of solution through the ports into the well over the array of nanostructures.

For multiplexing, 0.2uL of solution was spotted onto the 4 outer corners of the array (A, C, G and I) as shown in Supplementary Figure S1 (B). The surface chemistry of the solutions adhering to the gold surface allow such droplets to be formed – omitting any cross contamination between solutions.

To estimate the performance of the instrument and sensing substrate for a multiplexing application, we need to evaluate the noise performance and establish error limitations that will impact sensitivity and performance limits of the platform. Multiple measurements of water, followed by 2M $MgSO_4$, were performed hourly and used to estimate the measurement error and potential drifts when no changes are made in the fluidic chamber. A 2 M $MgSO_4$ solution was prepared in water. An initial reference measurement is taken using water pipetted into the fluidic well. An initial measurement was taken at 0 minutes, and every 30 minutes thereafter. After 3 hrs, the water is removed and the salt solution is added for measurements at 0 minutes, and every 30 minutes thereafter.

Supplementary Figure S2 (A and B) and Table S1, show that the largest maximum-minimum (max-min) range is of 0.3 nm or less for both water and salt. Hence, we estimate measurement error to ±0.15 nm for $\Delta\lambda$ of both peaks. Errors can be larger when solutions are exchanged and this may occur due to variations in incident light wave fronts, temperature and concentration gradients, or changes on the metal-liquid interface (electrical double layer) over the 3mm x 3mm measurement area that have been shown previously to affect plasmonic sensing. Another useful parameter to consider is the value S that represents spacing in the wavelength values between the two peaks. ΔS is the change in the spacing in comparison to the initial measurement and this has previously been used as an additional parameter to measure protein interactions at the surface. The large ~20 nm resonance shift due to $MgSO_4$ is accompanied by ΔS of 0.2-0.3 nm indicating a slight difference between Peak 1 and Peak 2 in terms of the sensitivity to refractive index changes. However, for proteins and buffer solutions, we expect resonance shifts to be an order smaller as the proteins form only a monolayer and the buffer solutions are far lower in salt concentration than the solutions used here. The differences in sensitivity, and hence ΔS, seen in the salt solutions would be near negligible when resonance shifts are in the order of ~2-5nm.

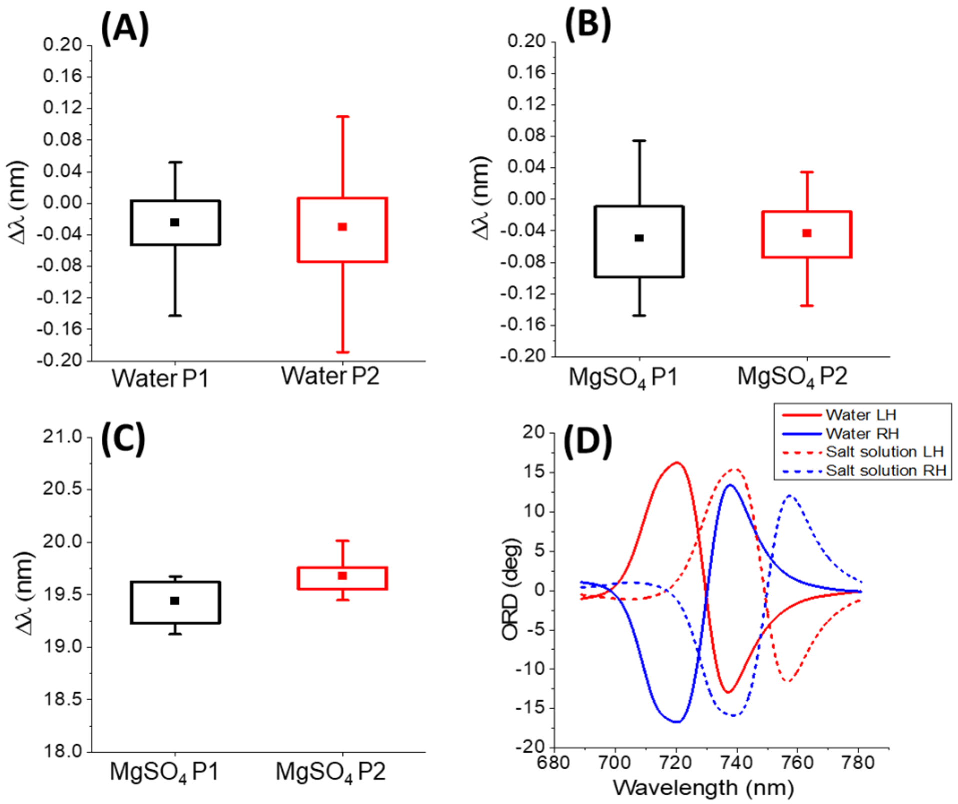

Supplementary Figure S2: (A) Resonance shifts (Δλ) with repeat water measurements and (B) repeat $MgSO_4$ measurements, over 3-hour periods. (C) Δλ obtained for $MgSO_4$ relative to water, with (D) change in ORD for both right- and left-handed structures. Boxplot whiskers show the max-min range of the data.

Values in nm	**Δλ of Water over 3hrs**		**Δλ of MgSO4 relative to Water**		**Δλ of MgSO4 over 3hrs**	
	**P1**	**P2**	**P1**	**P2**	**P1**	**P2**
**Mean Δλ**	-0.02	-0.03	19.44	19.68	-0.05	-0.04
**St.Dev. Δλ**	0.04	0.06	0.20	0.16	0.06	0.04
**Range Δλ**	0.19	0.30	0.55	0.57	0.22	0.17
**Mean ΔS**	-0.01	-	0.24	-	0.01	-
**St.Dev. ΔS**	0.07	-	0.21	-	0.07	-

Supplementary Table S1: Resonance shifts (Δλ) values of data shown in Figure 2. Values in nm.

Simulations for maximum electric field intensities

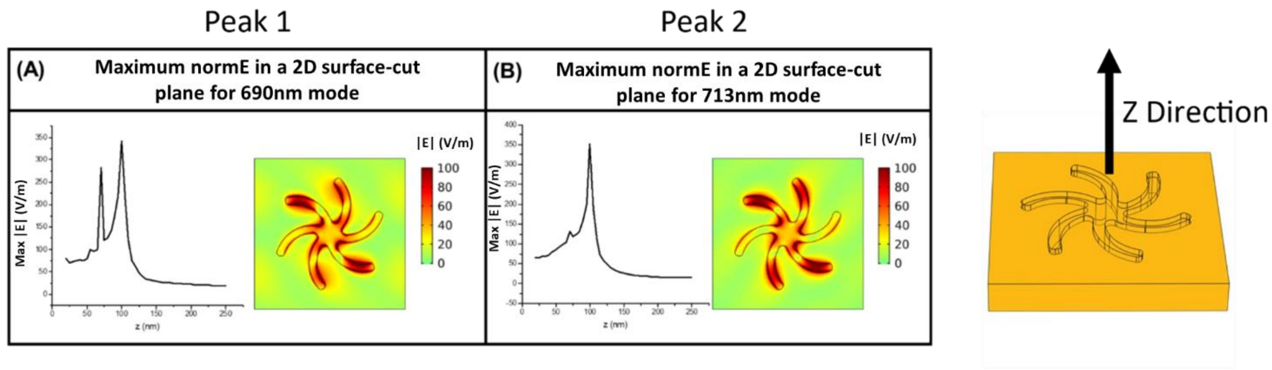

Supplementary Figure S3: Electric field magnitude, |E| (V/m) along z-axis above shuriken nanostructures for two separate modes: 690 nm (A) and 713 nm (B). Inset shows the 2D surface plots for |E| values on gold surface (z=100 nm) at these two modes.

The shuriken nanostructures were simulated using COMSOL 5.6. At the two peak positions, the electric field intensities were studied by measuring the maximum |E| field in the xy plane along the lateral z direction moving from the bottom of the indentation (at z=20 nm) up to 150 nm from the surface (at z=250 nm). For Peak 1 we see two lateral points that have the highest E fields, one inside the indentation and one on the surface. Peak 2 has a peak only at the surface. Both show that the |E| fields decay to <15% of the maximum by ~25 nm above the surface indicating sensitivity to material up to 20-30nm and showing high surface sensitivity, hence, the structures are not strongly affected by the bulk media.

Additional biosensing data

To evaluate whether non-specific binding is completely inhibited using spacer molecules, and whether the Biotin PEG thiol/spacer SAM is binding to the streptavidin as expected. An experiment was carried out where the spacer molecule alone was used to generate a SAM for complete passivation of any non-specific adsorption of proteins. The results showed that $MT(PEG)_4$ concentrations below 50mM in PBS leads to potential non-specific binding of streptavidin, as seen in Supplementary Figure S4, some weak fluorescence is observed at 10mM. However, this is completely omitted upon increasing the concentration to 100mM. Therefore, based on these results, future experiments were carried out using $MT(PEG)_4$ at a minimum concentration of 50mM. Bovine serum albumin (BSA) tagged with Alexa 647 was used as a control.

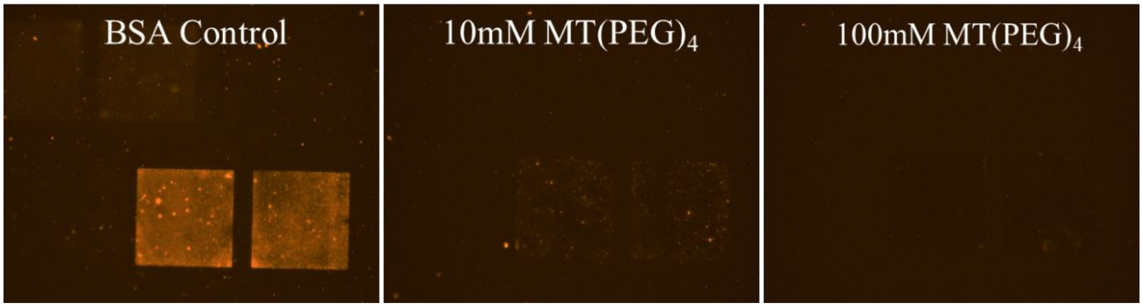

Supplementary Figure S4: Fluorescence Images of nanostructures with streptavidin Alexa 647 on surface, with varying spacer concentrations- 0, 10 and 100mM. BSA with Alexa 647 as control.

To test streptavidin biotin interactions, streptavidin (with Alexa 647 conjugate) is immobilised to the surface using a biotin PEG thiol SAM. Results for resonance shift measurements relative to initial water measurements are shown in Supplementary Figure S5 (A and B) and Supplementary Table S2.

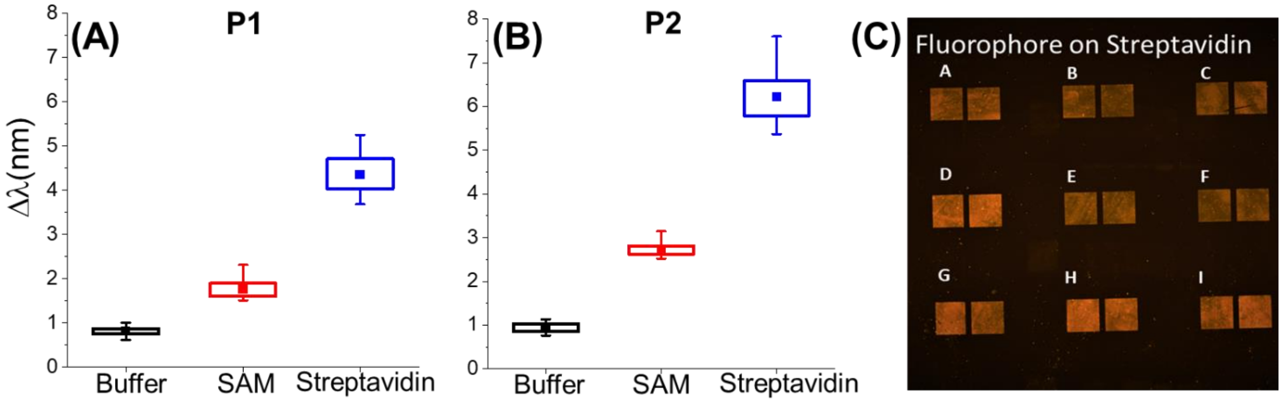

Supplementary Figure S5: Boxplot data for experiment with streptavidin conjugated with Alexa Fluor 647, binding to biotin PEG thiol SAM, for peak 1 (A) and peak 2 (B). Fluorescence from the streptavidin with Alexa Fluor 647 conjugate shown in (C). While protein is coating the entire surface, only the plasmonic enhanced fluorescence generates enough intensity to be visible hence the array pattern is visible.

The buffer solution shows an average resonance shift of 0.8 and 0.9 nm for P1 and P2 respectively, with ~0.1 nm standard deviation (σ) for both peaks. A SAM layer is then allowed to form onto the surface and rinsed with PBS. A mean $\Delta\lambda$ of 1.8nm is observed for P1 but P2 shifts more, 2.7nm on average. The σ values are still low even though the range is above 0.5nm showing relatively good homogeneity in the coverage over the entire surface. The SAM layer shows an increase in the spacing by 0.8 nm for $\Delta\lambda < 3$ nm. This is significantly large in comparison to the spacing change shown by the salt solutions. We attribute this change in spacing to a change in coupling of the optical modes as shown in previous work. (Kelly et al., 2018) Streptavidin is then immobilised onto the surface, rinsed with buffer after 1 hr exposure and then measured. P1 and P2 respectively show $\Delta\lambda$ of 4.4 nm and 6.2 nm, however, the larger σ values show a reduction in homogeneity of coverage, but such coverage is still relatively homogenous as also shown by fluorescence microscopy, Supplementary Figure S5 (C). ΔS values do not change indicating that the coupling may largely be affected by the SAM alone. The $\Delta\Delta\lambda$ values were also considered, however the σ values were matching or larger than the mean values in the case of SAM and streptavidin indicating a lack of statistical relevance. It is likely that this is a consequence of random orientations of the immobilised streptavidin and its own structure having overall higher symmetry in comparison to previously tested proteins such as enzymes and bovine serum albumin.

Values in nm	**Buffer**		**SAM**		**Streptavidin**	
	**P1**	**P2**	**P1**	**P2**	**P1**	**P2**
**Mean $\Delta\lambda$**	0.8	0.9	1.8	2.7	4.4	6.2

**St.Dev. Δλ**	0.1	0.1	0.2	0.2	0.4	0.6
**Range Δλ**	0.4	0.4	0.8	0.6	1.6	2.2
**Mean ΔS**	0.1	-	1.0	-	1.9	-
**St.Dev. ΔS**	0.1	-	0.2	-	0.3	-

Supplementary Table S2: Statistical information related to, Exp 1. All values in nm.

In another experiment, unconjugated streptavidin is immobilised to the surface using the same protocol as in Exp1. An additional step is performed where biotin conjugated with Atto 655 is added to bind to the streptavidin. Results are shown in Supplementary Figure S6 and Supplementary Table S3. The results are discussed in the main text.

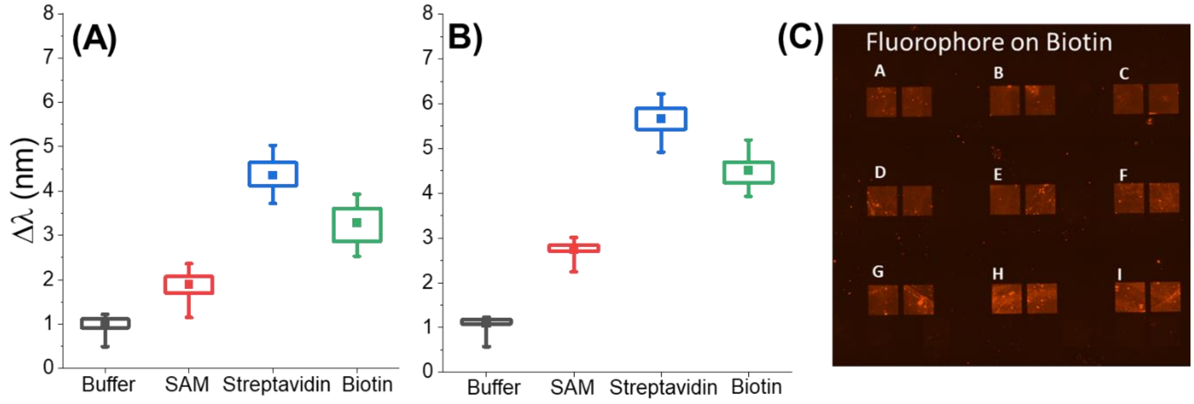

Supplementary Figure S6: Results from the second experiment, non-fluorescent streptavidin is used and an additional final step of binding biotin conjugated with Atto 655 to the streptavidin is performed, in which resonance shifts from peak 1 (A) and peak 2 (B) are also shown. (C) shows the fluorescence on the nanostructures from the biotin with Atto 655 conjugate. Initial measurements were taken in water and PBS, with all biosensing data taken relative to water. (D-F corresponds to Figure 3(B) in main text).

Values in nm	**Buffer**		**SAM**		**Streptavidin**		**Biotin**	
	**P1**	**P2**	**P1**	**P2**	**P1**	**P2**	**P1**	**P2**
**Mean Δλ**	1.0	1.1	1.9	2.7	4.4	5.7	3.3	4.5
**St.Dev. Δλ**	0.2	0.2	0.3	0.2	0.4	0.3	0.4	0.3
**Range Δλ**	0.7	0.7	1.2	0.8	1.3	1.3	1.4	1.3
**Mean ΔS**	0.1	-	0.9	-	1.3	-	1.2	-
**St.Dev. ΔS**	0.1	-	0.3	-	0.2	-	0.3	-

Supplementary Table S3: Statistical information related to Supplementary Figure S1, Experiment 2 (D-F). (Corresponds to Figure 3(B) in main text). All values in nm.

Emission and absorption spectra for Alexa 647 and Atto-655 (taken from ThermoFisher SpectraViewer) are shown in Supplementary Figure S7. For Alexa Fluor 647, the excitation peak lies at 653 nm whilst the

emission peak lies at 669 nm. In comparison, the maximum excitation wavelength (λex) for Atto 655 is 663nm, and the maximum emission wavelength (λex) is 684nm.

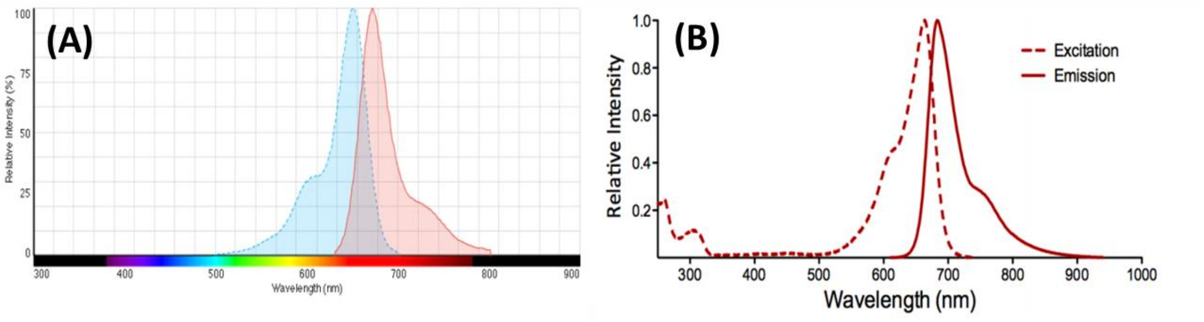

Supplementary Figure S7: Emission and Absorbance Spectra for Alexa Fluor 647 (A) and Atto-655 conjugate (B) (ThermoFisher SpectraViewer)

Additional data on multiplexing experiment for multi target diagnostics

The SARS-CoV-2 spike protein is a 1273 amino acid long (180-200 kDa) class I glycoprotein which can be cleaved into two subunits, spike 1 (S1) and spike 2 (S2), which play different roles in viral binding and entry into cells. For our experimental processes, the S1 subunit was used, which consists of an N-terminal domain (NTD; amino acid sequence 14-290), and a receptor binding domain (RBD; amino acid sequence 306-527). The 193-amino acid RBD constituent of the spike protein is responsible for recognising and binding to the angiotensin-converting enzyme-2 (ACE-2) on host cells. Prior to its use in a multiplexed DPA, we evaluated detection of anti-S1 antibodies in more realistic samples by mixing the antibodies in an artificially reconstituted mimic of human mucus, containing 0.2% mucin, plus 0.25 mg/ml haptoglobin and 0.50 mg/ml transferrin in phosphate buffer saline (PBS). The binders were tested against His-tagged recombinant S1-protein immobilised over the whole surface. Each measurement was taken relative to the SAM measurement after rinsing with PBS and Tween 20 solution to remove any non-specifically bound material on the surface, shown in Supplementary Figure S8. A mean Δλ value of 2.6 nm (Peak 2) was obtained for the successful immobilisation of the S1-protein onto the SAM. The artificial mucus was then applied for 15 minutes as a control to monitor any non-specific binding. Measurements with the artificial mucus itself shows non-specific binding with mean Δλ of 0.2 nm (Peak 2), values similar to resonance shift error (±0.3 nm) expected for changes in media performed previously. These are also much smaller than the expected shifts (>2nm) for anti-S1 antibodies (anti-S1 Ab) that are >100 kDa in mass. The artificial mucus was spiked with a 1 µM solution of anti-S1 Ab and applied to the biosensor for one hour in which positive shifts of 1.2nm (Peak 2) were observed for the change in the mean Δλ, portraying binding of the anti-S1 Ab to the S1 protein. From the data displayed in Supplementary Figure S8, there is minimal variance across the 9 structures, portrayed by the box plots (25-75% quartile shown by the box and max-min range by the whiskers) indicating a similar response from all arrays at each stage of the experiment. In particular, the anti-S1 Ab maximum and minimum data lies within a 0.3 nm range for Peak 2.

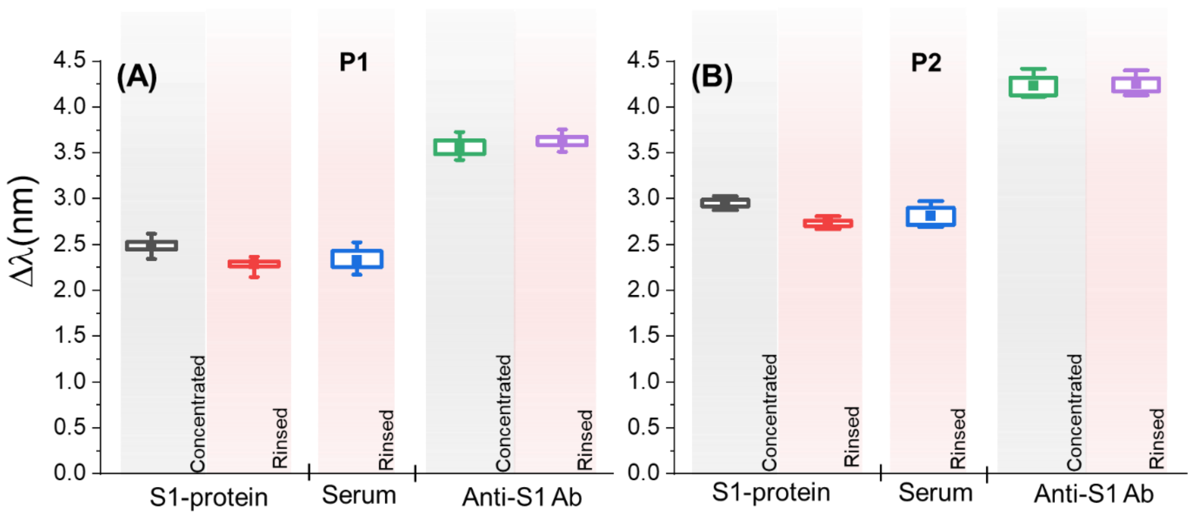

Supplementary Figure S8: Boxplot data for 1uM S1-Protein binding to pre-functionalised 1:4 NTA/EG-thiol SAM, followed by addition of artificial mucus, which was then spiked with 1µM S1-Antibody. Biosensing measurements taken relative to SAM. Peak 1 (A) and Peak 2 (B) rinsed data only. (Corresponds to Figure 4 (B) in main text where only rinsed results are used).

Values in nm	**S1-Protein Concentrated**		**S1-Protein Rinsed**		**Artificial Mucus Rinsed**		**S1-Antibody Concentrated**		**S1-Antibody Rinsed**	
	**P1**	**P2**	**P1**	**P2**	**P1**	**P2**	**P1**	**P2**	**P1**	**P2**
**Mean Δλ**	2.5	2.9	2.1	2.6	2.3	2.8	3.6	4.2	3.3	4.0
**St.Dev. Δλ**	0.1	0.1	0.2	0.1	0.2	0.1	0.1	0.1	0.1	0.1
**Range Δλ**	0.3	0.2	0.2	0.2	0.4	0.3	0.3	0.3	0.3	0.3

Supplementary Table S4: Data related to Figure 3(B) in main text.

In other experiments in which concentrated and rinsed measurements of the artificial mucus were taken, it was found that there was minimal difference in the resonance shifts obtained between the two measurements. This implies that bulk refractive index has no effect because of the way the fields are situated relative to the sample as shown earlier by simulations showing a high degree of surface sensitivity.

Additional data on multiplexing experiment for multi target diagnostics

Data for S1-protein experiments without artificial mucus

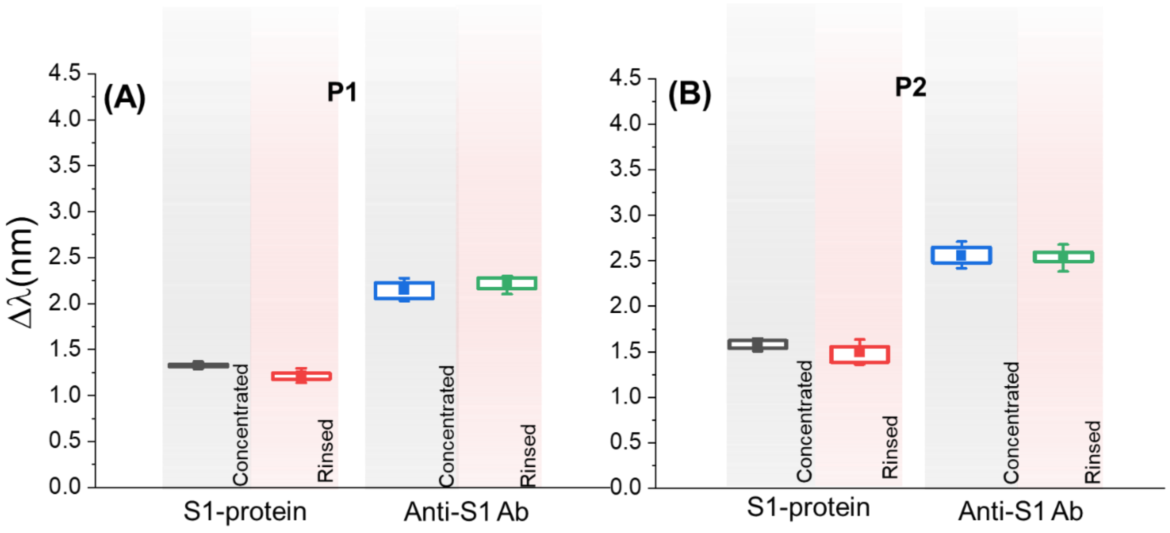

Supplementary Figure S9: Boxplot data for 1uM S1-Protein binding to pre-functionalised 1:4 NTA/EG-thiol SAM, followed by addition of 1uM S1-Antibody. Biosensing measurements taken relative to SAM. Peak 1 (A) and Peak 2 (B) concentrated and rinsed data.

Data for Multiplexing (S1, Streptavidin) - Related to Figure 4 (C) in main text.

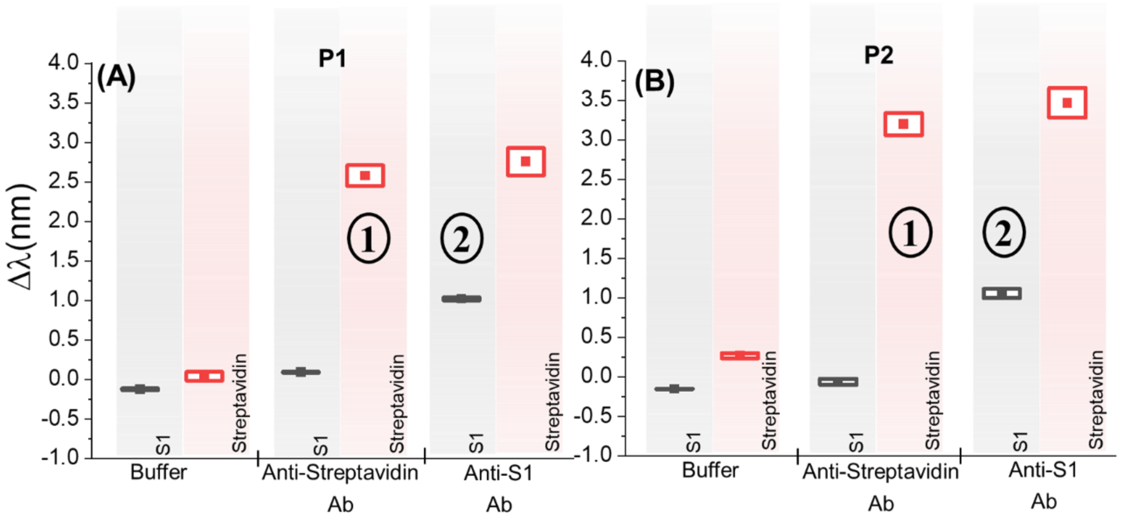

Supplementary Figure S10: Boxplot data for 1uM S1-Protein and 1uM streptavidin histagged spotted onto pre-functionalised SAM in multiplexing arrangement. Followed by addition of 1uM Anti-Streptavidin antibody then 1uM S1-Antibody. Biosensing measurements taken relative to proteins spotted onto SAM following a PBS rinse. Peak 1 (A) and Peak 2 (B) rinsed only data shown (corresponds to Figure 3 (C) in main text).

	Array G & I Buffer		Array A & C Buffer		Array G & I Step 1		Array A & C Step 1		Array G & I Step 2		Array A & C Step 2	
Values in nm	S1-Protein Tween		Streptavidin Tween		S1-Protein Anti-Streptavidin Antibody		Streptavidin Anti-Streptavidin Antibody		S1-Protein S1-Antibody		S1-Protein S1-Antibody	
	P1	P2	P1	P2	P1	P2	P1	P2	P1	P2	P1	P2
Mean Δλ	-0.1	-0.2	0.1	0.3	0.1	-0.1	2.6	3.3	1.0	1.1	2.8	3.5
St.Dev. Δλ	0.1	0.1	0.1	0.1	0.1	0.1	0.2	0.2	0.1	0.1	0.2	0.3
Range Δλ	0.2	0.1	0.3	0.3	0.3	0.2	0.5	0.6	0.3	0.3	0.6	0.7

Supplementary Table S5: Data related to Supplementary Figure S10. Rinsed data shown. Values in nm.

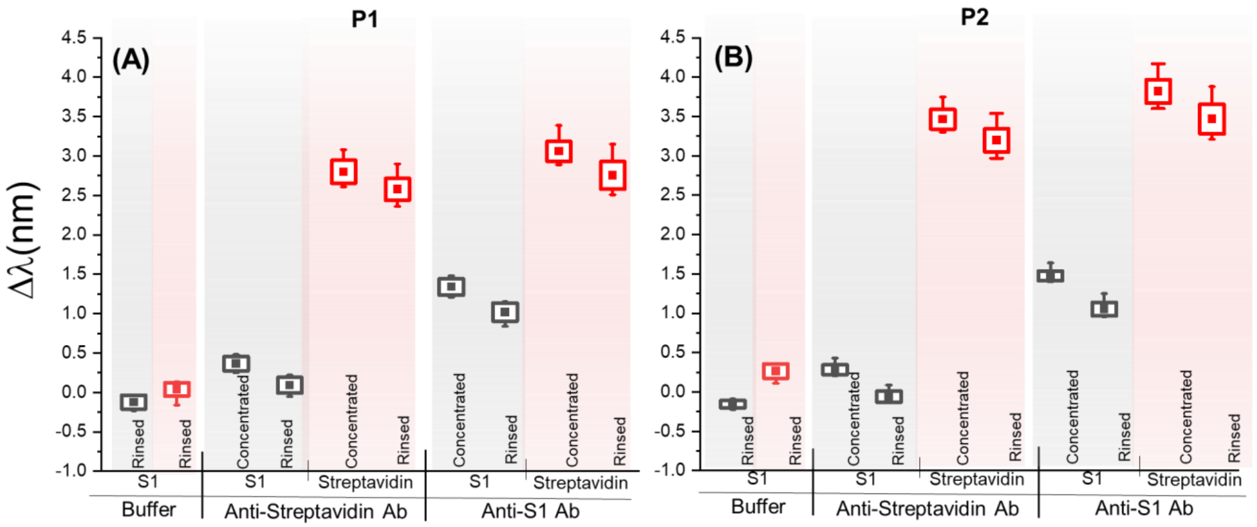

Supplementary Figure S11: Concentrated and rinsed data for Supplementary Figure S10 and Figure 3 (C) of main text for multiplexed detection of anti-S1 and anti-Streptavidin antibodies. Peak 1 (A) and Peak 2 (B) data shown for concentrated and rinsed measurements.

The sample was pre-functionalised with the NTA/EG-thiol SAM before each of the 2 proteins were spotted onto the sample as shown in

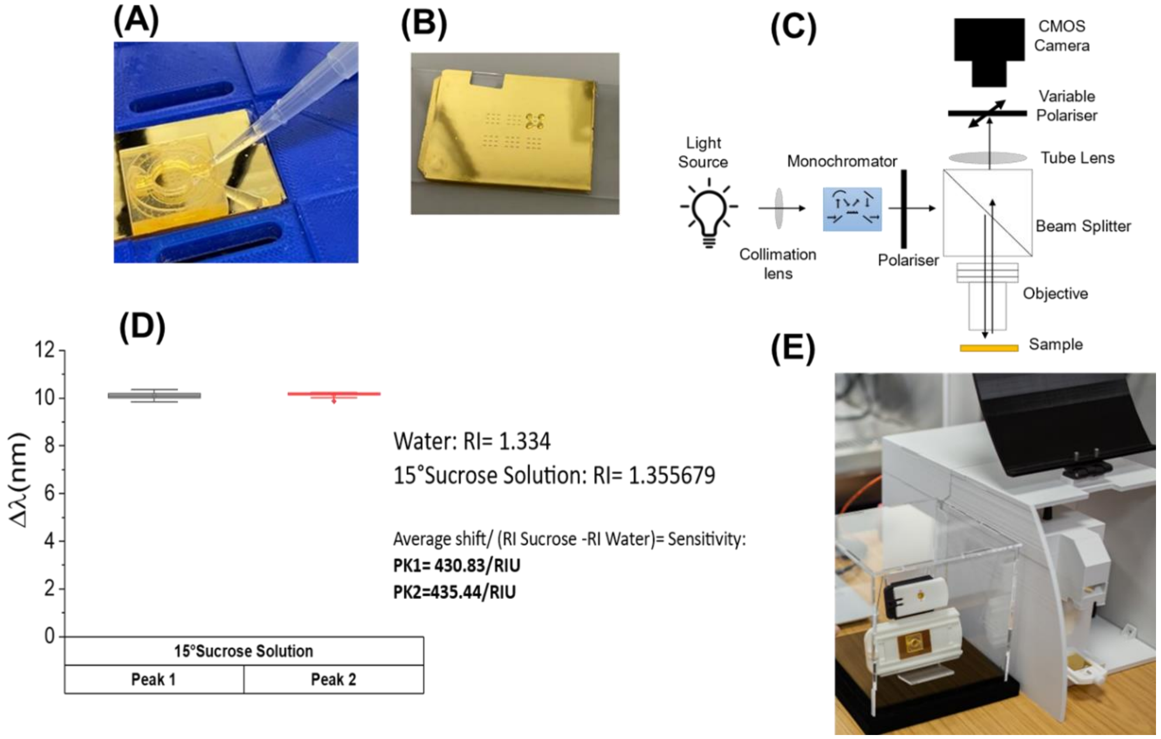

Supplementary Figure S1(B). In step 1, anti-streptavidin was introduced which caused minimal non-specific binding of this antibody to the S1-protein (0.1 nm P1 and -0.1 nm P2 which are smaller than the region of error). The streptavidin coated arrays show 2.6 nm for P1 and 3.3 nm for P2 indicating strong binding, Supplementary Table S5. Immobilisation of the anti-S1 in step 2 shows binding only to the S1-protein giving mean shifts of 1 nm for P1 and 1.1 nm for P2. Again, very minimal further shifts were obtained for the streptavidin coated arrays following rinsing (0.2 nm for P1 and P2).

Using antibodies to detect inactivated SARS-CoV-2 virus with plasmonic sensors

This nonspecific endopeptidase produces F(ab’)$_2$ fragments by enzymatically digesting the Fc portion of the IgG below the hinge region. The F(ab’)$_2$ fragments are made up of 2 Fab’ fragments that contain the antigen-binding sections of the antibody joined together by disulphide bonds. Upon addition of the F(ab’)$_2$ fragments to the Au surface, the disulphide bridges reduce to form F(ab’) fragments, in which the freed sulfhydryl groups facilitate attachment to the gold surface providing an attachment point for the antigens). Hence, the Fab’ fragment can be directly functionalised onto the TPS to develop a DPA for detection of SARS-CoV-2 viruses with the biosensor. Below are the results of the experiment described in Figure 4 of the main text.

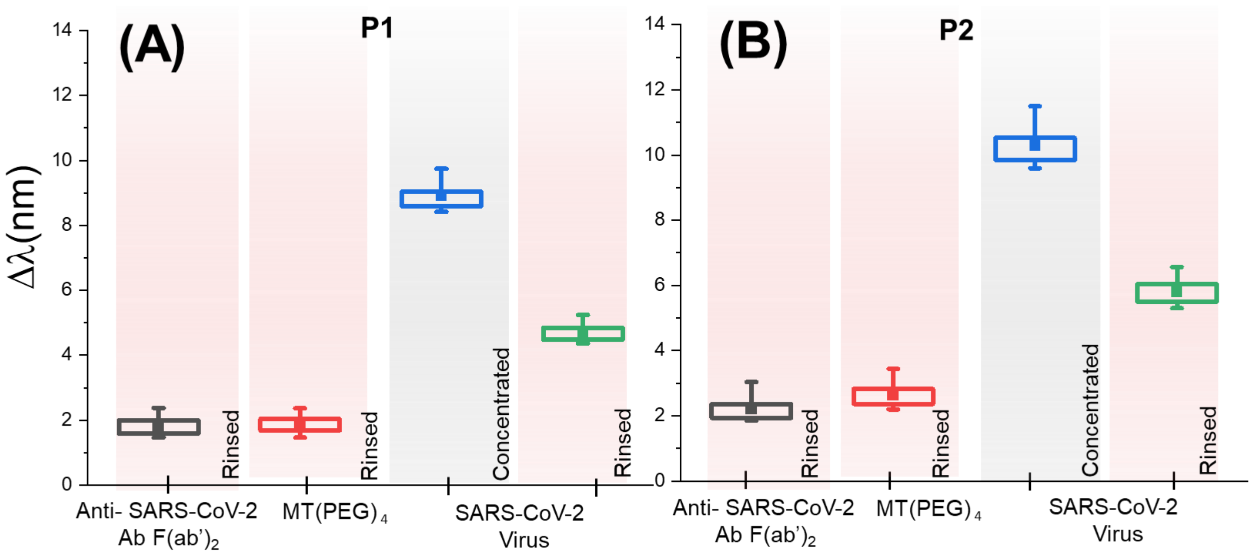

Supplementary Figure S12: Boxplot data for SAM functionalisation with Anti-SARS-CoV-2 Ab F(ab')$_2$ and 2mM MT(PEG)$_4$. Followed by addition of SARS-CoV-2 Virus in TRIS buffer. Biosensing measurements taken relative to an initial PBS rinsed measurement prior to functionalisation. No concentrated measurements were taken prior to addition of SARS-CoV-2 virus (corresponds to Figure 4 (B) in main text). Peak 1 (A) and Peak 2 (B) measurements shown.

Values in nm	**Anti-SARS-CoV-2 Ab F(ab')$_2$**		**MT(PEG)$_4$**		**SARS-CoV-2 Virus Concentrated**		**SARS-CoV-2 Virus Rinsed**	
	**P1**	**P2**	**P1**	**P2**	**P1**	**P2**	**P1**	**P2**
**Mean Δλ**	1.81	2.21	1.86	2.64	8.92	10.29	4.54	6.45
**St.Dev. Δλ**	0.31	0.41	0.31	0.43	0.51	0.71	2.38	3.54
**Range Δλ**	1.00	1.25	1.02	1.41	1.81	3.54	1.41	1.96

Supplementary Table S6: Statistical data related to Supplementary Figure S12. Values in nm.

Additional information on multiplexed DPA for virus detection

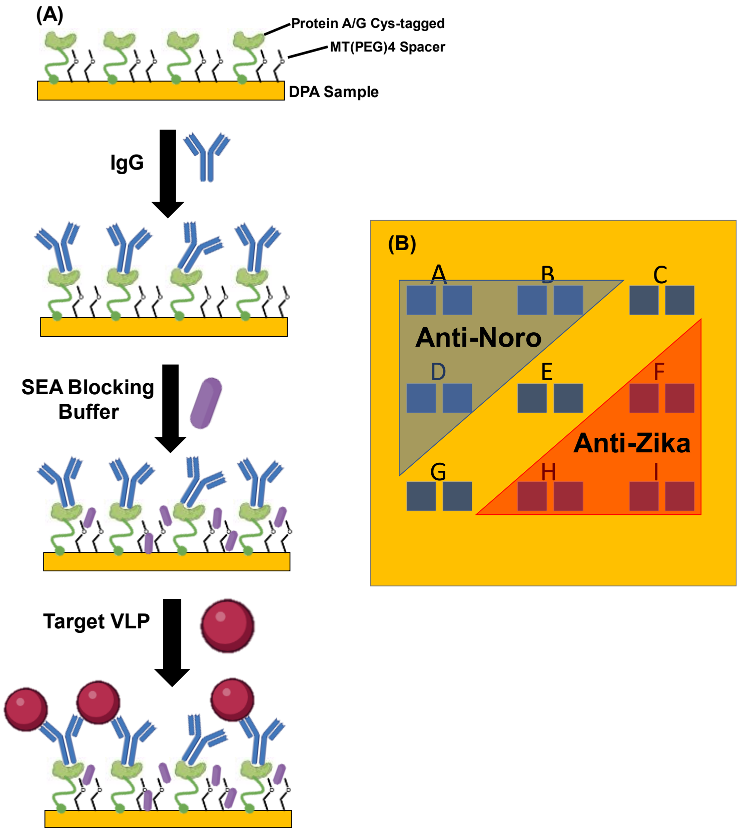

Supplementary Figure S13: (A) Scheme for functionalisation of Abs for multiplexed virus detection and (B) description of where specific antibodies were coated over the sample.

The bacterial cell wall fusion protein known as Protein A/G possesses a high affinity for IgG class antibodies. Equipped with a Cysteine-tag, this protein was utilised to functionalise a DPA sample with two different antibodies for a virus multiplexing experiment, with the general functionalisation scheme presented in Supplementary Figure S13 (A).

The Cys-tagged Protein A/G and the MT(PEG)$_4$ spacer SAM was prepared on a DPA in ultrapure water (details in main text). The concentration of Protein A/G was chosen to be 6.3 µM, or 0.3 mg ml^{-1}, and the MT(PEG)$_4$ spacer concentration was chosen relative to Protein A/G such that the final ratio would be 1:30, resulting in a 189 µM MT(PEG)$_4$ concentration. Prior to SAM application, a 2-well Ibidi cell culture insert was attached to the DPA sample with tweezers to isolate the regions shown in Supplementary Figure S13 (B). 50 µL of thiol solution was added to each well, and the sample left overnight in a sealed container for 16 hrs.

The sample was rinsed with PBS to remove any excess Protein A/G and spacer. Using the same Ibidi 2-well insert, 50 µL each of 1 µM concentrations of either anti-Norovirus or anti-Zika virus antibodies were added to their respective wells (Supplementary Figure S13 (B)) for 2 hrs to create regions with different specificities. The sample was rinsed with PBS to remove any excess unbound antibodies. SEA blocking buffer was added to both wells for 30 min in order to ensure any possible gaps in the SAM would be passivated and any non-specific interactions minimised. The sample was rinsed with PBS and the Ibidi 2-well insert removed with tweezers and a fluidic chamber attached. Due to the hydrophilic nature of the

functionalised SAM surface, some PBS remains during this process which ensures protein regions do not dry out. The fluidic chamber was filled with 100 µL PBS and a baseline measurement was taken.

Zika virus solution was then added to the fluidic chamber for 1 hr after which the sample was rinsed with PBS and measurements taken. Norovirus VLP solution was then added to chamber for 1 hr after which the sample was rinsed with PBS and the final measurements taken.

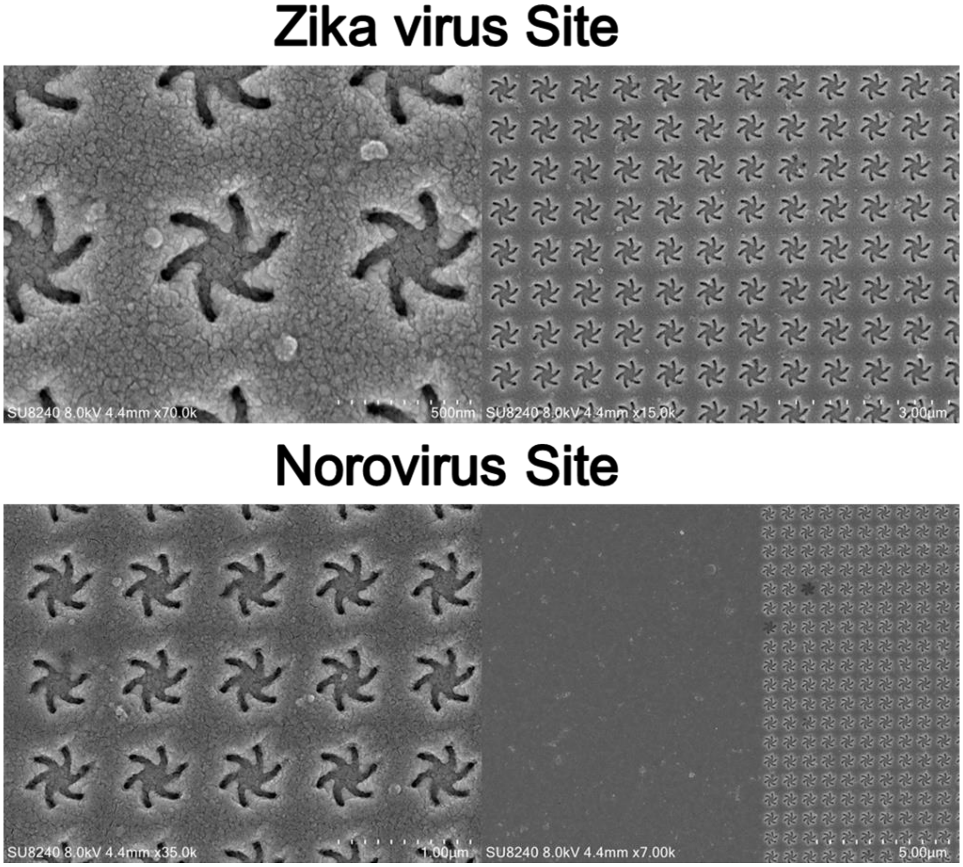

Supplementary Figure S14: SEM of final multiplexed virus DPA locations to show coverage by virus (or like) particles.

Limit of Detection (LoD)

For the LOD we performed concentration dependent measurements to complete a calibration curve. We selected Zika as the target for this assessment. Fluidic chambers were attached to four DPA samples, which were then prepared following the Protein A/G and MT(PEG)$_4$ spacer protocol (details in main text). All samples were rinsed with PBS, and anti-Zika virus mAb at 1 µM prepared in PBS was added to all four DPA samples for 2 hrs. The samples were rinsed with PBS, and SEA blocking buffer (Thermo 37527) added to all samples for 30 min. The samples were rinsed with PBS, and baseline measurements performed for each sample. Four ZIKV solutions of various concentrations were added to the samples for 1 h to investigate virus concentration dependency. The samples were rinsed with PBS and measurements for each sample performed.

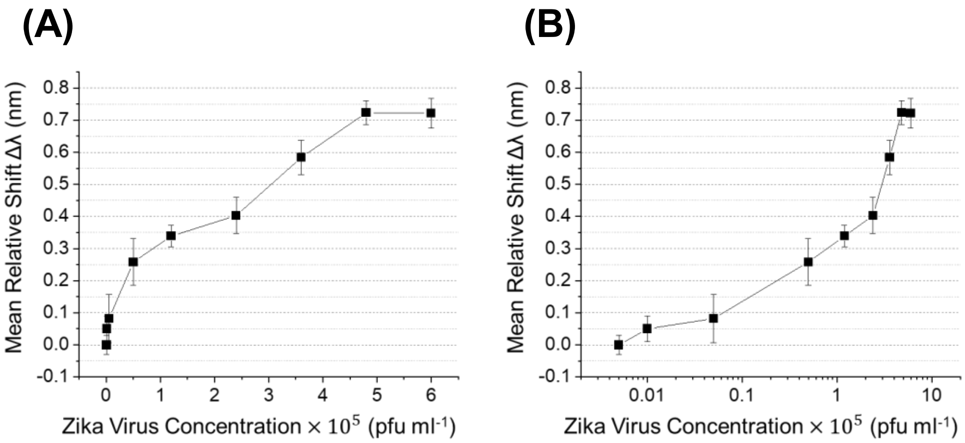

Supplementary Figure S15: ZIKV dilutions tested to estimate LoD with (A) linear and (B) logarithmic x-axis. Error bars represent standard error. Values used are from Peak 2.

The error bars in Supplementary Figure S15 represent standard error (SE). SE is calculated using the equation:

$$\frac{\sigma}{\sqrt{n}}$$

Where σ is the standard deviation, and n is the number of measurements. The measurements for each data set are from the 18 individual nanostructure arrays taken from a single experiment for each concentration.

LOD calculation can be performed using:

$$LOD = \frac{\bar{X}_{blank} + 3(\sigma_{blank})}{Slope_{linear}}$$

Where $\bar{X}$ is the mean of the blank measurements and σ_{blank} is the standard deviation of blank measurement. $Slope_{linear}$ is the slope of linear fit in the lower concentration region where the curve fits a linear regression. (Cennamo et al. 2021)

Using values from water tests in Table S1 for the blank measurements, we use 0.06 for σ_{blank} and using Supplementary Figure S15 (A), we evaluate the slope with the first four data points as 0.49 x 10^{-5}. This leads to a LOD of 3.1 x 10^4 pfu/ml.

References:

Kelly, C., Khosravi Khorashad, L., Gadegaard, N., Barron, L.D., Govorov, A.O., Karimullah, A.S., Kadodwala, M., 2018. Controlling Metamaterial Transparency with Superchiral Fields. ACS Photonics 5, 535–543.

Kötz, R.; Kolb, D. M.; Sass, J. K. Electron Density Effects in Surface Plasmon Excitation on Silver and Gold Electrodes. Surf Sci 1977, 69 (1), 359–364.

Wang, S.; Huang, X.; Shan, X.; Foley, K. J.; Tao, N. Electrochemical Surface Plasmon Resonance: Basic Formalism and Experimental Validation. Anal Chem 2010, 82 (3), 935–941.

Kelly, C.; Tullius, R.; Lapthorn, A. J.; Gadegaard, N.; Cooke, G.; Barron, L. D.; Karimullah, A. S.; Rotello, V. M.; Kadodwala, M. Chiral Plasmonic Fields Probe Structural Order of Biointerfaces. J Am Chem Soc 2018, 140 (27), 8509–8517.

Kelly, C.; Khosravi Khorashad, L.; Gadegaard, N.; Barron, L. D.; Govorov, A. O.; Karimullah, A. S.; Kadodwala, M. Controlling Metamaterial Transparency with Superchiral Fields. ACS Photonics 2017, 5 (2), 535–543.

Huang, Y.; Yang, C.; Xu, X.; Xu, W.; Liu, S. Structural and Functional Properties of SARS-CoV-2 Spike Protein: Potential Antivirus Drug Development for COVID-19. *Acta Pharmacol Sin* 2020, *41* (9), 1141–1149.

Mahmood, Z.; Alrefai, H.; Hetta, H. F.; A. Kader, H.; Munawar, N.; Abdul Rahman, S.; Elshaer, S.; Batiha, G. E.-S.; Muhammad, K. Investigating Virological, Immunological, and Pathological Avenues to Identify Potential Targets for Developing COVID-19 Treatment and Prevention Strategies. *Vaccines* 2020, *8* (3), 443.

Cennamo, N.; Pasquardini, L.; Arcadio, F.; Lunelli, L.; Vanzetti, L.; Carafa, V.; Altucci, L.; Zeni, L.; SARS-CoV-2 spike protein detection through a plasmonic D-shaped plastic optical fiber aptasensor, *Talanta*, 2021, *233*, 122532.
